# Assessment of Myocardial Function in Kenyan Children With Severe, Acute Malnutrition: The Cardiac Physiology in Malnutrition (CAPMAL) Study

**DOI:** 10.1001/jamanetworkopen.2019.1054

**Published:** 2019-03-22

**Authors:** Bernadette Brent, Nchafatso Obonyo, Samuel Akech, Mohammed Shebbe, Ayub Mpoya, Neema Mturi, James A. Berkley, Robert M. R. Tulloh, Kathryn Maitland

**Affiliations:** 1 Kenya Medical Research Institute Wellcome Trust Research Programme, Centre for Geographic Medicine Research-Coast, Kilifi, Kenya; 2 Department of Paediatrics, Faculty of Medicine, St Mary’s Campus, Imperial College, London, United Kingdom; 3 Centre for Tropical Medicine & Global Health, Nuffield Department of Medicine, University of Oxford, Oxford, United Kingdom; 4 Bristol Royal Hospital for Children, Bristol, United Kingdom

## Abstract

**Importance:**

Mortality among African children hospitalized with severe malnutrition remains high, with sudden, unexpected deaths leading to speculation about potential cardiac causes. Malnutrition is considered high risk for cardiac failure, but evidence is limited.

**Objective:**

To investigate the role of cardiovascular dysfunction in African children with severe, acute malnutrition (SAM).

**Design, Setting, and Participants:**

A prospective, matched case-control study, the Cardiac Physiology in Malnutrition (CAPMAL) study, of 88 children with SAM (exposed) vs 22 severity-matched patients without SAM (unexposed) was conducted between March 7, 2011, and February 20, 2012; data analysis was performed from October 1, 2012, to March 1, 2016.

**Exposures:**

Echocardiographic and electrocardiographic (ECG) recordings (including 7-day Holter monitoring) at admission, day 7, and day 28.

**Main Outcomes and Measures:**

Findings in children with (cases) and without (controls) SAM and in marasmus and kwashiorkor phenotypes were compared.

**Results:**

Eighty-eight children (52 with marasmus and 36 with kwashiorkor) of the 418 admitted with SAM and 22 severity-matched controls were studied. A total of 63 children (57%) were boys; median age at admission was 19 months (range, 12-39 months). On admission, abnormalities more common in cases vs controls included severe hypokalemia (potassium <2.5 mEq/L) (18 of 81 [22%] vs 0%), hypoalbuminemia (albumin level <3.4 g/dL) (66 of 88 [75%] vs 4 of 22 [18%]), and hypothyroidism (free thyroxine level <0.70 ng/dL or thyrotropin level >4.2 mU/L) (18 of 74 [24%] vs 1 of 21 [5%]) and were associated with typical electrocardiographic changes (T-wave inversion: odds ratio, 7.3; 95% CI, 1.9-28.0; *P* = .001), which corrected as potassium levels improved. Fourteen children with SAM (16%) but no controls died. Myocardial mass was lower in cases on admission but not by day 7. Results of the Tei Index, a measure of global cardiac function, were within the reference range and similar in cases (median, 0.37; interquartile range [IQR], 0.26-0.45) and controls (median, 0.36; IQR, 0.28-0.42). Echocardiography detected no evidence of cardiac failure among children with SAM, including those receiving intravenous fluids to correct hypovolemia. Cardiac dysfunction was generally associated with comorbidity and typical of hypovolemia, with low cardiac index (median, 4.9 L/min/m^2^; IQR, 3.9-6.1 L/min/m^2^), high systemic vascular resistance index (median, 1333 dyne seconds/cm^5^/m^2^; IQR, 1133-1752 dyne seconds/cm^5^/m^2^), and with few differences between the marasmus and kwashiorkor manifestations of malnutrition. Seven-day continuous ECG Holter monitoring during the high-risk initial refeeding period demonstrated self-limiting significant ventricular arrhythmias in 33 of 55 cases (60%) and 6 of 18 controls (33%) (*P* = .049); none were temporally related to adverse events, including fatalities.

**Conclusions and Relevance:**

There is little evidence that African children with SAM are at greater risk of cardiac dysfunction or clinically significant arrhythmias than those without SAM or that marasmus and kwashiorkor differed in cardiovascular profile. These findings should prompt a review of current guidelines.

## Key Points

**Question:**

Do high-risk African children with severe malnutrition show abnormal myocardial function consistent with biventricular failure and/or life-threatening arrhythmias?

**Findings:**

In a longitudinal case-control study of 88 children with severe, acute malnutrition, no evidence was found that malnourished children were at greater risk of cardiac dysfunction or clinically significant arrhythmias than nonmalnourished controls. Myocardial function was similar in the 2 marasmus and kwashiorkor clinical phenotypes, and myocardial indices following intravenous rehydration indicated fluid-responsive changes with no child developing fluid overload.

**Meaning:**

Research is needed to address current guidelines restricting rehydration for severe gastroenteritis to oral therapy.

## Teaser

This case-control study examines the risk for cardiac dysfunction in African children hospitalized for severe, acute malnutrition.

## Introduction

Worldwide, approximately 2.1 million children die annually as a direct consequence of malnutrition.^1,2^ Severe, acute malnutrition (SAM) remains a frequent cause of pediatric hospitalization in much of the developing world,^3^ where it is associated with in-hospital mortality rates as high as 20%^4^ and poor long-term outcomes^5,6^ despite adherence to World Health Organization (WHO) treatment guidelines.^4,5^ Factors underlying these poor outcomes are multifactorial but frequently involve comorbid complications including severe pneumonia, diarrhea, sepsis, and HIV.^4^

Over several decades, investigators have tried to determine the most common causes of death in children with SAM.^4,7,8^ Because many of these children die suddenly and unexpectedly, some have postulated that myocardial dysfunction, manifesting as cardiac arrhythmias or heart failure, may underpin these deaths.^9,10,11,12,13,14^ Severe, acute malnutrition is clinically rationalized into 2 phenotypes: marasmus, characterized by severe wasting, and kwashiorkor, characterized by bilateral pitting edema and other skin and hair changes, with or without severe wasting. There is a widely held belief that children with SAM, especially those with kwashiorkor,^14^ are at risk of imminent cardiac failure and sodium overload.^15^ This hypothesis has resulted in management guidelines discouraging any intravenous volume replacement in children with SAM, even in those with cholera.^16^ Evidence underpinning this guideline is weak,^17^ mainly derived from observational studies^14,18^ without concomitant assessments of clinical status or relevant physiological investigations. Both nutritional status and comorbidities could independently affect cardiac function, increasing the risk of cardiac dysfunction. However, it is unclear to what extent each factor contributes to any perturbations in myocardial function without a systematic, multiparametric longitudinal assessment. The Cardiac Physiology in Malnutrition (CAPMAL) study describes cardiac function and electrocardiographic (ECG) findings during nutritional rehabilitation in children with SAM.

## Methods

We conducted a prospective, matched case-control study comparing outcomes of patients with SAM (exposed) with the outcomes of severity-matched patients without malnutrition (unexposed) conducted between March 7, 2011, and February 20, 2012; data analysis was performed from October 1, 2012, to March 1, 2016. Children presenting to the Kilifi District Hospital in Kilifi, Kenya, between Monday and Thursday from 8 am and 8 pm were screened for study eligibility. Enrollment was suspended once all the 3 ECG Holter monitors were in use. Eligible children fulfilled WHO criteria for SAM^15^ (cases), defined as marasmus (weight-for-length *z* score <−3 of the WHO growth standard, or a mid-upper arm circumference measurement of <11.5 cm) or kwashiorkor (bilateral nonpitting edema with or without severe wasting). Enrollment was consecutive until at least 25 children with kwashiorkor and 50 children with marasmus were recruited. To compare interpretation of changes in cardiac physiological status, nonmalnourished children, who were frequency matched (4 cases to 1 control) for age, sex, and admission syndrome (controls), were systematically enrolled. Clinical definitions for the admission-presenting syndromes (eg, severe pneumonia, gastroenteritis) were based on WHO criteria.^15^ Children with congenital heart disease were excluded.

Full written informed parental consent was obtained for all participants. The Kenyan Medical Research Institute/National Ethical Review Committee approved the study. Participants received travel reimbursement. This study followed the Strengthening the Reporting of Observational Studies in Epidemiology (STROBE) reporting guideline.

At admission and days 7 and 28 after admission, both groups underwent structured clinical, anthropometric, laboratory, echocardiographic, and ECG assessments. Standard investigations at admission included a hemogram, malaria film, blood cultures, and HIV antibody testing. Stored plasma was used for retrospective analysis of clinical chemistry and thyroid function. A computer-generated random subset of patients was assessed for vitamin D and N-terminal pro brain natriuretic peptide levels, as defined by Nir et al,^19^ using electrochemiluminescence immune assay by Meso Scale Discovery.

Participants with gastroenteritis and features of impaired perfusion received intravenous rehydration if they fulfilled the European Pediatric Life Support criteria for shock comprising lethargy or unconsciousness, temperature gradient, weak pulse, tachycardia, and prolonged capillary refill time (>2 seconds).^20^ Intravenous rehydration was administered as a fluid bolus of Ringer lactate, 20 mL/kg/h, and repeated no more than 2 times if the patient still had 2 or more signs of shock and no clinical signs of pulmonary edema. No intravenous correction of electrolyte deficits was prescribed.

### Echocardiography and ECGs

Echocardiograms were performed at set time points using a portable echocardiograph (Vivid.i 6S-RS or 10S-RS phased array transducer; GE Healthcare), following American Society of Echocardiography guidelines.^21,22^ For each measurement, an average of 3 consecutive readings was calculated. These readings were reviewed independently by a cardiologist (R.M.R.T.) blinded to participant group and clinical status. Twelve-lead ECGs were recorded digitally (CardioSoft v6.51 Diagnostic System; GE Healthcare) following American Heart Association guidelines.^23^ For data interpretation, published cutoff ranges for age and sex were applied^24^ (eMethods in the Supplement) and variables were stratified for body surface area (BSA).

### Holter Monitoring

We hypothesized that early deaths may be associated with arrhythmias that develop during refeeding in children, including those with SAM, at high risk of phosphate depletion.^25,26^ We therefore studied a random subset of cases and controls (always ensuring that 1 of the 3 monitors was free to use on the next participant after admission) on a 3-lead continuous ECG monitoring system for 7 days (Lifecard CF Holter monitor; Spacelabs) and analyzed data with Pathfinder SL Digital software, version 8.0 (Spacelabs Healthcare, 2011), including standardized arrhythmia criteria. We defined significant ventricular arrhythmias as couplets, triplets, salvos, R-on-T, and ventricular tachycardias.

### Statistical Analysis

Because the major concern for children with SAM at risk of cardiac failure principally relates to the kwashiorkor rather than marasmus phenotype,^14^ we hypothesized that there would be no difference in cardiac function between these clinical phenotypes and estimated that a sample size of at least 25 children with the kwashiorkor phenotype and 50 children with the marasmic phenotype would provide greater than 90% power to detect a 15% relative intergroup difference in mean ejection fraction and fractional shortening.

Categorical variables were compared by Pearson χ^2^ analysis and proportions by logistic regression, reported as odds ratios (ORs) with 95% CIs. The Wilcoxon rank sum test for nonparametric data or a 2-sided, unpaired *t* test for normally distributed data were used for intergroup comparisons of continuous variables between the groups (eMethods in the Supplement). We present crude ORs and 95% CIs for associations between putative clinical risk factors and indices of both cardiac function and death. To identify independent associations between these exposures and outcomes, we used multivariable logistic regression and present adjusted ORs and 95% CIs. We included in each model all variables associated with the outcome in the univariable analysis and/or those with a strong a priori hypothesis of an association.^27^ All analyses were performed using Stata, version 10 (StataCorp).

## Results

During the course of the study, 418 children were admitted with SAM, from whom we enrolled 88 children with SAM (cases; 52 with the marasmus and 36 with the kwashiorkor phenotype) and 22 nonmalnourished children (controls). A total of 63 children (57%) were boys; median age at admission was 19 months (range, 12-39 months). Baseline demographics, as well as clinical and laboratory findings, are summarized in Table 1. Cases often presented with 1 or more major comorbidity (including HIV in 20 of 88 children [23%]); however, the syndromic matching with controls resulted in few differences between the groups, which were principally pneumonia, diarrhea, and sepsis. Notable in cases vs controls was a higher frequency of biochemical abnormalities, which included severe hypokalemia (potassium level <2.5 mEq/L [to convert to millimoles per liter, multiply by 1]) in 18 of 81 (22%) vs 0%; hypoalbuminemia (albumin level <3.4 g/dL [to convert to grams per liter, multiply by 10]) in 66 of 88 (75%) vs 4 of 22 (18%), and biochemical indicators of hypothyroidism (free thyroxine level <0.70 ng/dL [to convert to picomoles per liter, multiply by 12.871] or thyrotropin level >4.2 mU/L) in 18 of 74 (24%) vs 1 of 21 (5%) children.

**Table 1.** Baseline Characteristics and Outcomes of Children With and Without SAM

Characteristic	SAM Cases (n = 88)	Controls (n = 22)	*P* Value
Male, No. (%)	48 (55)	15 (68)	.25
Age, median (IQR), mo	19 (13 to 35)	19 (12 to 39)	.68
Anthropometry			
Mid-upper arm circumference, median (IQR), cm	10.8 (10 to 11.5)	14.9 (13.5 to 16.0)	<.001
Weight-for-height *z* score, median (IQR)	−3.2 (−3.8 to −2.5)	−1.1 (−1.6 to −0.5)	<.001
Temperature, No. (%)			
Hyperthermia (>37.5°C)	25 (28)	7 (32)	.75
Hypothermia (<35.0°C)	2 (2)	0	.48
Respiratory findings, No. (%)			
Tachypnea^a^	18 (20)	10 (46)	.02
Indrawing	5 (6)	3 (14)	.20
Deep breathing	6 (7)	3 (14)	.30
Hypoxemia (SpO_2_ <95%)	1 (1)	1 (5)	.28
Cardiovascular findings, No. (%)			
Tachycardia^b^	7 (8)	2 (9)	.86
Impaired perfusion^c^	15 (17)	2 (9)	.36
Hypotension^d^	8 (9)	0	.14
Gallop rhythm	2/87 (2)	0/21	.48
Hepatomegaly^e^	15 (17)	2 (9)	.36
Neck vein distention	1/87 (1)	0	.61
Hydration status, No. (%)			
Decreased skin turgor^f^	8/81 (10)	0/21	.13
Sunken eyes	22/85 (26)	2 (9)	.09
Dry mucous membranes	10/84 (12)	1 (5)	.31
Altered conscious level	11 (13)	4 (18)	.49
Major clinical syndromes, No. (%)			
Pneumonia (WHO criteria, any severity)^g^	23 (26)	6 (27)	.91
Diarrhea (≥3 loose stools/24 h)	16 (18)	6 (27)	.34
Cerebral palsy	10 (11)	0	.10
Sepsis (SIRS criteria)^h^	29 (33)	11 (50)	.14
Hematology, No. (%)			
Severe anemia (hemoglobin <5 g/dL)	2 (2)	1 (5)	.56
Leukocytosis ^h^	21 (24)	7 (32)	.44
Infection, No. (%)			
Malaria parasites present	2/75 (3)	3/13 (23)	.003
HIV antibody present	20 (23)	0	.01
Clinical biochemistry, No. (%)			
Hyponatramia (sodium <125 mEq/L)	4/81 (5)	0/18	.34
Hypokalemia (potassium <2.5 mEq/L)	18/81 (22)	0/18	.03
Low (corrected) calcium (<8.4 mg/dL)	6 (7)	1 (5)	.70
Low magnesium (<1.5 mEq/L)	4 (5)	1 (5)	>.99
Hypophosphatemia (<2.5 ng/mL)	9 (10)	0	.12
Hypoalbuminemia (albumin <3.4 g/dL)	66 (75)	4 (18)	.001
Severe hypoalbuminemia (albumin <1.5 g/dL)	14 (16)	0	.045
High creatinine (>6542 mg/dL)	5 (6)	0	.25
High urea nitrogen (>17.9 mg/dL)	8 (9)	3 (14)	.53
Hypothyroidism^i^	18/74 (24)	1/21 (5)	.048
Acidosis, No. (%)			
Acidemia (pH <7.2)	10/79 (13)	2/19 (11)	.80
Base deficit (>−10 mEq/L)	27/72 (38)	6/19 (32)	.63
Cardiac markers, No. (%)			
High lactate (>27 mg/dL)	15/82 (18)	4 (18)	.99
NT-pro BNP elevation^j^	18/47 (38)	4/10 (40)	.92
Cardiac mass, median (IQR), g			
LVM	23 (17 to 31)	33 (26 to 47)	<.001
LVM indexed to height	50 (42 to 62)	60 (51 to 71)	.009
LVM indexed to body surface area	63 (52 to 77)	71 (57 to 93)	.03
Outcome, No. (%)			
Death at any time	14 (16)	0	.05
Late deaths (>7 d in hospital)	11/85 (13)	0	.11

Abbreviations: IQR, interquartile range; LVM, left ventricular mass; NT-pro BNP, N-terminal pro brain natriuretic peptide; SAM, severe, acute malnutrition; SIRS, systemic inflammatory response syndrome; SpO_2_, peripheral capillary oxygen saturation; WHO, World Health Organization.

SI conversion factors: To convert albumin to grams per liter, multiply by 10; calcium to millimoles per liter, multiply by 0.25; creatinine to micromoles per liter, multiply by 88.4; hemoglobin to grams per liter, multiply by 10; lactate to millimoles per liter, multiply by 0.111; magnesium to millimoles per liter, multiply by 0.5; phosphate to millimoles per liter, multiply by 0.323; potassium to millimoles per liter, multiply by 1; sodium to millimoles per liter, multiply by 1; and urea nitrogen to millimoles per liter, multiply by 0.357.

^a^ Respiratory rate at 50 breaths/min or more (age 2-11 months), 40 breaths/min or more (age 1-5 years), and 30 breaths/min or more (age >5 years).

^b^ Heart rate greater than 180 beats/min (age <12 months) and greater than 160 beats/min (age 1-5 years).

^c^ Any one of tachycardia, prolonged capillary refill time, weak pulse, or temperature gradient.

^d^ Systolic blood pressure less than 70 mm Hg (age <12 months), less than 70 mm Hg + (2 × age in years) (age 1-10 years), and less than 90 mm Hg (age >10 years).

^e^ Palpable liver edge 2 cm or more below the costal margin.

^f^ Return of skin pinch of longer than 2 seconds.

^g^ Signs of mild or severe pneumonia according to WHO criteria.

^h^ Both SIRS and leukocytosis were defined per Goldstein criteria.^28^

^i^ Either free thyroxine less than 0.70 ng/dL (to convert thyroxine to picomoles to per liter, multiply by 12.871) or thyrotropin greater than 4.2 mU/L.

^j^ As defined by Nir et al.^19^

Hypoalbuminemia was particularly more prevalent in kwashiorkor (34 [94%]) than marasmus (32 [62%]) (*P* < .001). Hypophosphatemia (phosphate level <2.5 ng/mL [to convert to millimoles per liter, multiply by 0.323]) was not more common in cases (9 [10%]) than controls (none) (*P* = .12). Shock signs and hypotension (reported as impaired perfusion) were more common among cases than controls (15 [17%] vs 2 [9%]) but comparable in frequency between the marasmic and kwashiorkor phenotypes (*P* = .36). At admission, N-terminal pro brain natriuretic peptide concentrations were elevated, but comparable in cases and controls, and this balance persisted in both groups to day 28. Only 1 child, with culture-proven tuberculosis and pericardial effusion, required diuretics and captopril to treat cardiac failure, resulting in good recovery. By day 7, many abnormal factors had resolved, except for persistent severe hypoalbuminemia in 19 of 85 patients (22%) and metabolic acidosis (base deficit >10) in 25 of 76 children (33%) (eTable 1 and eTable 2 in the Supplement).

### ECG Findings

All children with SAM were in sinus rhythm at each time point (admission day 7 and day 28). The SAM cases had lower voltages than controls at all 3 time points plausibly related to the lower cardiac mass (Table 2; eTable 3 in the Supplement). Unique to SAM cases at admission were prolonged PR intervals (14 of 88 [16%]) and T-wave inversions in leads V5 (17 of 88 [19%]) and V6 (13 of 88 [15%]), largely associated with severe hypokalemia (T-wave inversion: OR, 7.3; 95% CI, 1.9-28.0; *P* = .001), which corrected as potassium levels improved. No U waves were seen. Short QTc intervals were present in 24 of 88 children with SAM (27%) at admission, persisting to day 7. ST depression was evident in 8 of 88 SAM cases (9%) at admission, with hypophosphatemia (phosphate level <2.5 mg/dL) as the major risk factor (OR, 25.8; 95% CI, 1.6-428; *P* < .001), although hypophosphatemia was uncommon, occurring in 9 of 88 (10%) on admission and 6 of 80 (8%) at day 7. Low limb-lead voltages (<0.5 mV) were present in 34 of 88 children with SAM (39%) on admission, 24 of 83 (29%) on day 7, and 11 of 61 (18%) on day 28, with total voltage and serum albumin level correlated. For every 1-g/L increase in serum albumin concentration, voltage increased by 0.2 mV (Spearman correlation coefficient, *r* = 0.37; *P* < .001). Although we found a number of ECG abnormalities in children with SAM, with most corrected by day 7, none of the abnormal admission ECG patterns were temporally associated with death (11 of 14 deaths [79%] occurred after day 7).

**Table 2.** Comparison of 12-Lead ECG Findings in SAM Cases and Controls at Admission, Day 7, and Day 28

ECG Measure^a^	Admission			Day 7			Day 28		
	SAM Cases (n = 88)	Controls (n = 22)	*P* Value	SAM Cases (n = 83)	Controls (n = 18)	*P* Value	SAM Cases (n = 62)	Controls (n = 19)	*P* Value
Heart rate, median (IQR), beats/min	132 (120-142)	141 (127-156)	.04	133 (124-148)	128 (110-140)	.06	134 (121-146)	126 (114-139)	.07
HRV, median (IQR), ms	25 (15-45)	15 (10-25)	.05	25 (15-50)	28 (15-45)	.93	30 (20-45)	40 (20-60)	.26
P axis, median (IQR)	53 (40-62)	53 (40-62)	.54	45 (36-56)	44 (34-53)	.88	45 (36-56)	49 (37-53)	.18
QRS axis, median (IQR)	66 (35-86)	57 (37-77)	.40	55 (28-78)	50 (28-72)	.80	59 (40-80)	60 (34-75)	.89
RAD, No. (%)	2 (2)	0	.48	2 (2)	0	.52	2 (3)	0	.43
LAD, No. (%)	10 (11)	2 (9)	.76	9 (11)	2 (11)	.51	9 (15)	2 (11)	.66
T axis, median (IQR)	31 (3.5-54)	40 (25-51)	.21	36 (17-51)	42 (30-51)	.28	34 (20-47)	35 (31-54)	.36
Abnormal T axis, No. (%)	20 (23)	2 (9)	.15	7 (8)	0	.20	4 (6)	0	.27
QRS-*t* >90°, No. (%)	16 (18)	0	.03	5 (6)	0	.29	4 (6)	0	.26
PR interval, median (IQR), ms	115 (106-131)	112 (107-127)	.79	109 (102-125)	132 (121-134)	.002	112 (103-127)	125 (117-131)	.006
Short PR interval, No. (%)	2 (2)	0	.48	2 (2)	0	.51	0	0	NA
Long PR interval, No. (%)	14 (16)	1 (5)	.20	5 (6)	2 (11)	.44	2 (3)	1 (5)	.68
QRS duration, median (IQR), ms	67 (60-74)	66 (62-72)	.96	60 (55-67)	66 (62-73)	.002	60 (56-66)	64 (61-73)	.007
Short QRS duration, No. (%)	6 (7)	1 (5)	.70	26 (31)	0	.006	16 (26)	1 (5)	.09
Long QRS duration, No. (%)	3 (3)	0	.38	0	0	NA	0	0	NA
QTc interval, median (IQR), ms	413 (381-431)	42 (409-437)	.05	401 (383-417)	434 (421-445)	<.001	417 (403-430)	430 (417-444)	.02
Short QTc interval, No. (%)	24 (27)	1 (5)	.02	20 (24)	0	.02	7 (11)	1 (5)	.44
Long QTc interval, No. (%)	3 (3)	0	.38	0	1 (6)	.03	1 (2)	0	.59
Inverted T wave at V4 lead, No. (%)	20 (23)	0	.01	8 (9)	0	.18	10 (16)	0	.06
Inverted T wave at V5 lead, No. (%)	17 (19)	0	.03	4 (5)	0	.35	3 (5)	0	.33
Inverted T wave at V6 lead, No. (%)	13 (15)	0	.06	3 (4)	0	.42	3 (5)	0	.29
ST depression, No. (%)	8 (9)	1 (5)	.46	2 (2)	0	.51	1 (2)	0	.58
ST elevation, No. (%)	1 (1)	0	.62	0	0	NA	5 (8)	0	.20

Abbreviations: ECG, electrocardiogram; HRV, heart rate variability; IQR, interquartile range; LAD, left axis deviation; NA, not applicable; RAD, right axis deviation; SAM, severe, acute malnutrition.

^a^ Cutoff levels were derived using definitions by Semizel et al.^24^

### Holter Monitoring

Holter monitoring was conducted on 55 children with SAM and 18 controls. The median duration of continuous ECG monitoring was less in the controls because of their shorter hospital stays (38 vs 64 hours; *P* < .001). Median (interquartile range [IQR]) hourly minimum and maximum heart rates demonstrated higher heart rates in children with SAM vs controls, with minimum rates of 117 beats/min (IQR, 104-128 beats/min) vs 111 beats/min (IQR, 98-124 beats/min) and maximum rates of 145 beats/min (IQR, 133-159 beats/min) vs 137 beats/min (IQR, 124-151 beats/min). A total of 6831 hours of continuous ECG monitoring were analyzed in SAM cases. Overall, 13 bradycardic episodes (heart rate <45 beats/min for ≥4 beats) were recorded in 3 children with SAM but none in controls. Significant ventricular arrhythmias were present in 33 of 55 SAM cases (60%) and 6 of 18 controls (33%) (*P* = .049), with a mean of 26 vs 22 episodes per 24 hours, respectively; all were asymptomatic and self-limiting (eTable 4 in the Supplement). Although 5 of 55 patients (9%) with SAM died before day 28, we found no association between significant ventricular arrhythmias and death (OR, 2.9; 95% CI, 0.3-28.0; *P* = .36).

### Echocardiographic Findings

#### Cardiac Structure and Mass

At admission, children with SAM had reduced absolute left ventricular mass relative to controls—a difference that was less pronounced after indexing (Table 1). By day 7, these differences were nonsignificant, with median BSA index masses of 58 g (IQR, 47-70 g) and 70 g (IQR, 54-88 g), respectively (*P* = .66) (eTable 1 in the Supplement). Similarly, median values for echocardiographic variables, when corrected using published reference ranges and stratified by BSA,^29^ exhibited few differences between cases and controls (eFigure 1 and eTable 6 in the Supplement). Relative to children with marasmus, those with kwashiorkor had a lower left ventricular mass index at admission (45 vs 53 g/m^2.7^; *P* = .004) and on day 7 (52 vs 65 g/m^2.7^; *P* = .004) (eTable 7 in the Supplement). Small, asymptomatic pericardial effusions (<0.5 cm) were present in 18 of 88 (20%) of the cases (eTable 8 in the Supplement) and were more prevalent in kwashiorkor than marasmus phenotypes (11 of 36 [31%] vs 7 of 52 [14%]; *P* = .05), possibly associated with severe hypoalbuminemia (OR, 4.2; 95% CI, 1.0-18.3; *P* = .06). In marasmus cases, pericardial effusions were associated with biochemical markers of hypothyroidism (OR, 6.0; 95% CI, 1.2-30.7; *P* = .03).

#### Cardiac Function

**Figure 1.**
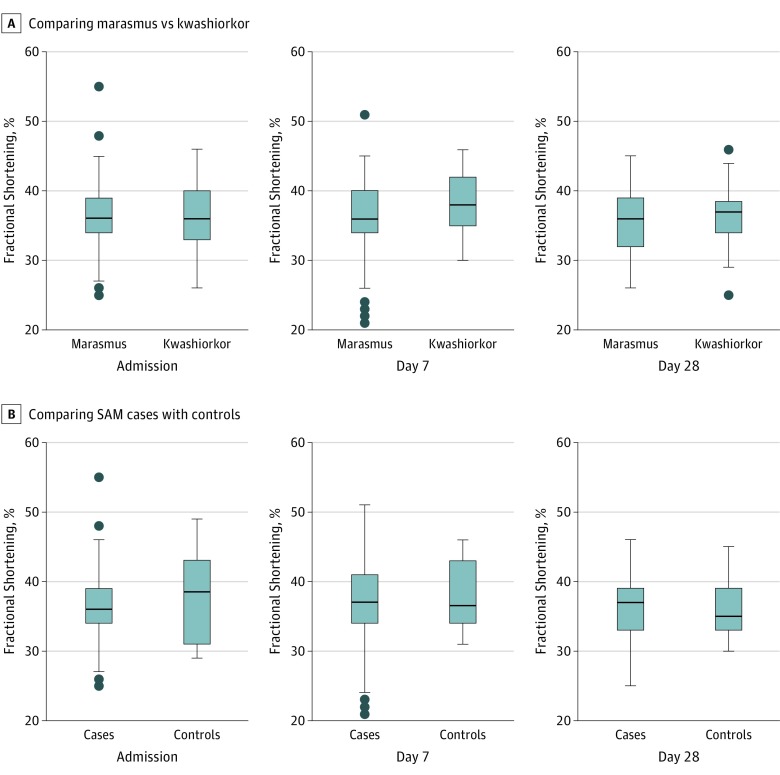
Cardiac Fractional Shortening Indices at Admission, Day 7 and Day 28 Cardiac fractional shortening indices over time in marasmus vs kwashiorkor phenotypes (A) and children with (cases) and without (controls) severe, acute malnutrition (B). The horizontal line in the middle of each box indicates the median, while the top and bottom borders of the box mark the 75th and 25th percentiles, respectively. The whiskers above and below the box mark the 90th and 10th percentiles. The points beyond the whiskers are outliers. SAM indicates severe, acute malnutrition.

We found no evidence that children with SAM were more likely to have overall systolic, diastolic, or global cardiac dysfunction than controls (Table 3). The Tei Index values, a measure of global cardiac function, were within the reference range and similar in cases (median, 0.37; IQR, 0.26-0.45) and controls (median, 0.36; IQR, 0.28-0.42) at admission. This similarity suggests that the abnormalities were associated with the underlying comorbidity rather than with malnutrition per se. Contrary to our hypothesis, we found no difference in fractional shortening between children with marasmus and kwashiorkor (Figure 1), with measurements similar to those of controls across all time points (Table 3; eTable 9 and eTable 10 in the Supplement). Cardiac dysfunction was generally associated with comorbidity and typical of hypovolemia, with low cardiac index (median, 4.9 L/min/m^2^; IQR, 3.9-6.1 L/min/m^2^) and a high systemic vascular resistance index (median, 1333 dyne seconds/cm^5^/m^2^; IQR, 1133-1752 dyne seconds/cm^5^/m^2^) (Table 3). Systolic function was more affected in association with marasmus (day 7 only) with low fractional shortening (6 of 48 [13%] vs 0%; *P* = .03) and diastolic function (low early diastolic filling velocities) more prevalent in kwashiorkor (3 of 34 [9%] vs 0%; *P* = .049) (eTable 9 and eTable 10 in the Supplement). Risk factors for systolic dysfunction were HIV (OR, 3.6; 95% CI, 1.1-11.1; *P* = .02) and systemic inflammatory response syndrome (OR, 3.6; 95% CI, 1.2-10.6; *P* = .01), with no significant differences between marasmus and kwashiorkor cases (χ^2^ for heterogeneity >0.5).

**Table 3.** Cardiac Function Indices at Admission Comparing Children With SAM vs Controls and Children With Marasmus vs Kwashiorkor

Value at Admission	SAM Cases (n = 88)	Controls (n = 22)	*P* Value	Marasmus (n = 52)	Kwashiorkor (n = 36)	*P* Value
**Systolic**						
FS, median (IQR), %	36 (34-39)	38.5 (31-43)	.28	36 (34-39)	36 (33-40)	.73
High, No./total No. (%)	5/83 (6)	4/22 (18)	.19	4/48 (8)	1/35 (3)	.30
Low, No./total No. (%)	6/83 (7)	0/22	.19	4/48 (8)	2/35 (6)	.65
EF, median (IQR), %	68 (65-73)	71 (61-75)	.5	69 (65-73)	68 (64-72)	.72
MAPSE, median (IQR), %	8 (7-10)	10 (9-12)	<.001	13 (11-15)	14 (13-16)	.12
High, No./total No. (%)	5/81 (6)	3/22 (14)	.25	4/49 (8)	1/32 (3)	.36
Low, No./total No. (%)	8/81 (10)	2/22 (9)	.91	5/49 (10)	3/32 (9)	.90
TAPSE, median (IQR), %	14 (12-16)	16 (14-20)	<.001	8 (7-9)	9 (8-10)	.07
High, No./total No. (%)	3/82 (4)	0/22	.36	3/49 (6)	0/33	.15
Low, No./total No. (%)	3/82 (4)	0/22	.36	3/49 (6)	0/33	.15
**Diastolic**						
Ea, median (IQR)	1.17 (1.06-1.35)	1.18 (1.06-1.27)	.47	1.15 (1.08-1.37)	1.18 (1.04-1.35)	.97
E/E’, median (IQR), m/s	6.78 (5.75-8.29)	7.24 (6.32-8.29)	.35	7.28 (6.12-8.55)	6.08 (5.09-7.33)	.02
High, No./total No. (%)	0/78	1/19 (5)	.04	0/45	0/33	NA
Low, No./total No. (%)	1/78 (1)	0/19	.62	0/45	1/33 (3)	.24
**Global Function**						
Tei Index, median (IQR)	0.37 (0.26-0.45)	0.36 (0.28-0.42)	.59	0.38 (0.30-0.48)	0.30 (0.24-0.42)	.12
High, No./total No. (%)	20/82 (24)	3/22 (14)	.28	20/48 (42)	6/34 (18)	.23
**Hemodynamics**						
SV, median (IQR), mL	14 (10-18)	21 (17-25)	<.001	14 (10-18)	14 (11-18)	.65
SVI, median (IQR), mL/m^2^	39 (29-45)	37 (32-48)	.35	40 (31-46)	38 (27-44)	.44
High, No./total No. (%)	5/83 (6)	4/22 (18)	.07	4/48 (8)	1/35 (3)	.30
Low, No./total No. (%)	27/83 (33)	7/22 (32)	.95	14/48 (29)	13/35 (37)	.44
CO, median (IQR), L/min	1.8 (1.5-2.4)	2.7 (2.2-3.9)	<.001	1.7 (1.5-2.3)	1.8 (1.4-2.5)	.91
CI, median (IQR), L/min/m^2^	4.9 (3.9-6.1)	5.5 (4.8-7.2)	.05	5.2 (4.1-6.3)	4.5 (3.8-6.1)	.18
High, No./total No. (%)	15/83 (18)	7/22 (32)	.16	10/48 (21)	5/35 (14)	.44
Low, No./total No. (%)	14/83 (17)	1/22 (5)	.14	7/48 (15)	7/35 (20)	.52
SVR, median (IQR), dyn s/cm^5^	495 (431-594)	363 (309-402)	<.001	526 (435-696)	472 (428-524)	.08
SVRI, median (IQR), dyn s/cm^5^/m^2^	1333 (1133-1752)	676 (622-911)	<.001	1561 (1274-1841)	1237 (1040-1350)	.002
High, No./total No. (%)	28/81 (35)	0/21	.002	22/47 (47)	6/34 (18)	.006
Low, No./total No. (%)	5/81 (6)	13/21 (62)	<.001	2/47 (4)	3/34 (9)	.40

Abbreviations: CI, cardiac index; CO, cardiac output; Ea, early diastolic mitral annular velocities; E/E´, ratio of mitral peak velocity of early filling to early diastolic mitral annular velocity; EF, ejection fraction; FS, fractional shortening; IQR, interquartile range; MAPSE, mitral annular plane systolic excursion; NA, not applicable; SAM, severe, acute malnutrition; SV, stroke volume; SVI, SV index; SVR, systemic vascular resistance; SVRI, SVR index; TAPSE, tricuspid annular plane systolic excursion.

#### Hemodynamic Profiles

Vital signs were similar in cases and controls at all times, apart from children with SAM having a wider pulse pressure at admission (eFigure 2 in the Supplement). Compared with controls, cases had significantly lower median cardiac index, which was concurrent with a higher systemic vascular resistance index (SVRI) (Table 3). In addition, relative to controls, median SVRI in children with SAM was increased at all points to day 28 (Table 3; eTable 9 and eTable 10 in the Supplement). In support of improved intravascular filling,^30^ the left ventricular inner diameter in diastole *z* score increased over time (eFigure 1 and eTable 5 in the Supplement) in the absence of abnormally high left ventricular filling pressures (E/E’ ratio) (Table 3) in children with SAM compared with controls.

#### Responses to Fluid Expansion

Fifteen episodes of intravenous fluid resuscitation or intravenous rehydration were recorded involving 12 children with SAM (75% had marasmus). In the majority (10 of 12 [83%]), fluid was administered for hypovolemia secondary to profuse watery diarrhea (eTable 11 in the Supplement). All participants had an appropriate physiological response to fluid resuscitation for shock except 2 children who had sepsis and neutropenia. One patient had 3 episodes of hypovolemia during admission and received intravenous fluid rehydration for each as shown in eTable 11 in the Supplement.

**Figure 2.**
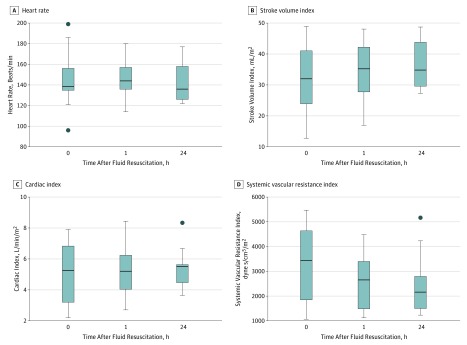
Cardiac Indices Before and After Intravenous Fluid Resuscitation in Severely Malnourished Children Results of intravenous fluid therapy for heart rate (A), stroke volume index (B), cardiac index (C), and systemic vascular resistance (D). The horizontal line in the middle of each box indicates the median, while the top and bottom borders of the box mark the 75th and 25th percentiles, respectively. The whiskers above and below the box mark the 90th and 10th percentiles. The points beyond the whiskers are outliers.

Echocardiographic indices were recorded before and at 1, 8, and 24 hours after fluid therapy. These results revealed high SVRI and low cardiac index initially, with some resolution by 24 hours (Figure 2; eTable 12 in the Supplement). Individual case responses are presented as Starling dose-response curves, which largely indicated an appropriate physiological response to fluid resuscitation in most instances (eFigure 3 in the Supplement). Stroke volume index and cardiac index increased slightly, whereas heart rate and SVRI fell in response to fluids. Of the 12 children who received fluid resuscitation for shock, 8 (67%) ultimately died. In 3 children, death occurred within 24 hours of fluid therapy despite having shown appropriate physiological response to fluid resuscitation (eTable 12 in the Supplement). No child developed biventricular failure or pulmonary edema, including children who died.

#### Mortality

By day 28, 14 of 88 children (16%) with SAM had died (8 with marasmus), with most deaths (11 [79%]) occurring after day 7; none of the children in the control group died. Children who died were more severely wasted (weight-for-height *z* score, −3.9 vs −3.1; *P* = .04) and experienced less weight gain. Those who died had a lower median left ventricular inner diameter in diastole *z* scores (−1.0 vs −0.2; *P* = .001), significantly higher median Tei Index scores (OR, 7.9; 95% CI, 1.7-26.3), a higher index of global systolic and diastolic left ventricular dysfunction (0.46 vs 0.33; *P* = .03), and lower mean cardiac index (3.75 vs 5.15 L/min/m^2^; *P* = .009) at admission than children with SAM who survived. Admission SVRI was similar in survivors and nonsurvivors (1325 vs 1565 ds/cm^5^/m^2^; *P* = .13). Apart from heart rate variability, no ECG abnormalities were associated with mortality, which was under 25 milliseconds in fatal cases. As a predictor of survival among children with SAM, a day 7 receiver operating characteristic curve for heart rate variability had an area under the curve of 0.9 with a predictive value for death of 27% (IQR, 12%-42%). Clinical details on the deaths are summarized in eTable 13 in the Supplement.

## Discussion

Clinical, echocardiographic, and ECG features of cardiac dysfunction were present in children with SAM but were not more prevalent than in nonmalnourished controls matched for presenting clinical syndromes. As demonstrated previously,^18,31,32,33^ we found evidence of reduced myocardial mass. However, when adjusted for BSA,^34^ few significant differences relative to reference values were evident. Similarly, the prevalence of abnormal cardiac function indices, when stratified by BSA, was proportionate to published cutoff levels. Together, the presence of a wide pulse pressure, evidence of systolic dysfunction, and hemodynamic features of low cardiac index and high SVRI indicated underfilling. Certain 12-lead ECG changes in SAM cases were largely temporally associated with electrolyte perturbations, protein status, and decreased muscle mass but were not clinically significant in our cohort. This conjecture is supported by the result of Holter monitoring over the first 7 days of admission, which, despite longer periods of recording in patients with SAM, did not show that malnourished children were more prone to arrhythmias than matched controls. Moreover, all observed arrhythmias were self-limiting, clinically silent, and identified retrospectively only during Holter analysis. Severe hypoalbuminemia was associated with an increased rate of small, functionally irrelevant pericardial effusions, particularly in children with kwashiorkor. In addition, when considering children with SAM with kwashiorkor and comparing with findings in the marasmus phenotype, we found a significant difference in left ventricular mass index, but no differences were evident when considering global myocardial function.

Starvation induces a cascade of hormonal alterations, leading to a reduced metabolic rate controlled by the hypothalamic-pituitary-thyroid axis. This condition has often been described as an adaptive hypocirculatory state of hypothyroidism, which manifests as a reduced ejection fraction, high SVRI, decreased blood volume, and, when chronic, a pericardial effusion (myxedema). Consistent with this state, we found low free thyroxine levels in 20% of the children with SAM, which may partly explain the persistence of raised SVRI and small pericardial effusions.^35^ The echocardiographic and hemodynamic parameters in children with abnormal myocardial function pointed toward hypovolemia rather than biventricular cardiac failure. The median cardiac index was lowest at admission, which, because other load-independent myocardial functional parameters were within reference ranges, makes underfilling the most likely interpretation. This underfilling or a decrease in blood volume is supported by a low median left ventricular inner diameter in diastole *z* score (a putative marker of preload) at admission and an elevated SVRI (indicating reduced intravascular filling). Furthermore, the low albumin state observed in the children with SAM, which possibly affected plasma oncotic pressure, might have contributed to intravascular fluid depletion.

Our work supports that of Viart,^18^ who studied intravascular volumes as well as cardiac and intravascular pressures using isotopes, dye dilutions, and catheters in Congolese children with SAM during nutritional rehabilitation. His findings indicated significantly reduced intravascular volume, reduced cardiac indices, and frank circulatory failure.

Contrary to current guideline recommendations,^15^ in the children in our study, both clinical and echocardiographic findings indicated largely fluid-responsive changes, without evidence of fluid overload. We suggest that these findings be cautiously interpreted in children with SAM with septic shock because high-quality evidence from the Fluid Expansion as a Supportive Therapy trial indicated harm from fluid bolus therapy in children without SAM,^36^ which is likely to be generalizable to those with SAM. For children with hypovolemia secondary to severe diarrhea, a 2017 study described similar fluid-responsive findings in children with SAM that was managed with a more liberal rehydration protocol.^37^ Nevertheless, although outcomes in children in this subgroup remain poor over the course of hospitalization, fatal events were probably unrelated in terms of time or event type to fluid resuscitation or rehydration.

Current WHO guidelines recommend avoiding administration of intravenous fluids in children with SAM, even for management of severe dehydration; however, these guidelines are based on expert opinion rather than empirical evidence and remain controversial.^17,38^ While a number of studies have identified low cardiac mass in children with SAM, other studies suggest relative cardiac sparing.^31,33,39^ In the largest study, which evaluated baseline cardiovascular function in 272 stable Malawian children, no significant differences were detected for cardiac index, stroke volume index, or heart rate between inpatient children with and without SAM.^40^

### Limitations

The major limitation of the present study is the relatively small number of controls (all HIV negative). Nevertheless, to our knowledge, this study is the first to attempt to match cases with SAM to controls by clinical syndrome. Through an in-depth and comprehensive investigation, the findings established similar perturbations in myocardial and hemodynamic physiological status in cases and controls, indicating that these abnormalities were unlikely to be attributable to the nutritional status or phenotype rather than the underlying comorbidity. Although we believe our study is the first to include Holter monitor data, for financial and logistical reasons, we were able to do continuous ECG monitoring only on a subset of children over the first week of admission during what is considered a critical period of refeeding (including several children with severe hypophosphatemia).^41^ We also were unable to continue monitoring for the full 4-week observation period. Nonetheless, we found no evidence of clinically relevant arrhythmias, even during the periadmission period when both severe electrolyte imbalance and ECG changes were prevalent.

We suggest that the addition of continuous ECG monitoring still represents an important advance from previous ECG studies of children with malnutrition, all of which have been limited to 12-lead ECG data. Furthermore, the median weight-for-height *z* score (−3.1) and case fatality rate (9%) of children who were fitted with a Holter monitor are roughly similar to the total population of severely malnourished children admitted to Kilifi District Hospital during the study period (weight-for-height *z* score, −3.1 and case fatality rate, 11%) (eTable 14 in the Supplement).

The presenting clinical syndromes, HIV prevalence (23%), and case mortality rate (16%) of children with SAM in the CAPMAL study are similar to other hospital SAM cohorts in Africa. The higher case mortality among participants receiving intravenous fluid for shock resuscitation corresponds to previously published data showing higher mortality rates in children with SAM with diarrhea and hypovolemia.^8^ Consequently, we believe that our findings are generalizable.

## Conclusions

To our knowledge, this is the first study to challenge the widely held perception that the heart of a malnourished child is at incipient risk of heart failure and significant arrhythmias. We found that perturbations of myocardial function were secondary to underlying complications or comorbidities, and there were few differences between the kwashiorkor and marasmic phenotypes. In addition, our data suggest largely fluid-responsive changes in children with SAM who receive rehydration. This finding challenges the current WHO management guidelines; research is needed to address the guidelines.

## Notes

Supplement. **eMethods.** Detailed Methodology **eTable 1.** Day 7 Clinical and Laboratory Findings Between Severely Malnourished (SAM) Children and Controls **eTable 2.** Day 28 Clinical and Laboratory Findings Between Severely Malnourished (SAM) Children and Controls **eTable 3.** Comparison of Mean R/S Voltage Between Children With Severely Malnourished (SAM) Cases and Controls at Admission, Day 7 and Day 28 **eTable 4.** Comparison of Total Number of Arrhythmias and Number of Affected Severely Malnourished (SAM) Cases and Controls **eTable 5.** Summary on Haemodynamic Indices Supporting Figure 1 **eTable 6.** Comparison of Cardiac Dimensions Represented as Raw Values and z-Scores Between Children With Marasmus and Kwashiorkor at Admission, Day 7 and Day 28 **eTable 7.** Comparing Median Left Ventricular Mass and Left Ventricular Mass Indices Between Those With Marasmus and Kwashiorkor at Admission, Day 7 and Day 28 **eTable 8.** Prevalence of Pericardial Effusions Among Malnourished Children Overall and Comparison Between Children With Marasmus and Kwashiorkor at Admission, Day 7 and Day 28 **eTable 9.** Cardiac Function Indices in Malnourished Children Overall and Controls and Children With Marasmus and kwashiorkor at Day 7 **eTable 10.** Cardiac Function Indices in Malnourished Children Overall and Controls and Children With Marasmus and Kwashiorkor at Day 28 **eTable 11.** Bedside Observations and Laboratory Findings in Severely Malnourished Participants With Shock at Onset of Fluid Resuscitation/Rehydration **eTable 12.** Summary of Changes in Cardiovascular Parameters in Response to Fluid Resuscitation/Rehydration **eTable 13.** Causes of Death and Relationship to Intravenous Fluid Therapy for the 14 Severely Malnourished Children Who Died **eTable 14.** Baseline Characteristics Between Recruited and Non-Recruited Severely Malnourished Children at Admission **eFigure 1.** z-Scores in Cases and Controls of Cardiac Dimensions at Admission (Day 0), Day 7 and Day 28 **eFigure 2.** Vital Features Over Time in Cases and Controls **eFigure 3.** Frank Starling Diagram of Individual Malnourished Children Pre- And Post-Fluid Resuscitation **eReferences**
