# Supplementary material for: Assessment of Myocardial Function in Kenyan Children With Severe, Acute Malnutrition: The Cardiac Physiology in Malnutrition (CAPMAL) Study
Source: JAMA Netw Open. 2019 Mar 22;2(3):e191054. doi: 10.1001/jamanetworkopen.2019.1054 (PMC6583281; doi:10.1001/jamanetworkopen.2019.1054)
Supplement: Supplement. — eMethods. Detailed Methodology eTable 1. Day 7 Clinical and Laboratory Findings Between Severely Malnourished (SAM) Children and Controls eTable 2. Day 28 Clinical and Laboratory Findings Between Severely Malnourished (SAM) Children and Controls eTable 3. Comparison of Mean R/S Voltage Between Children With Severely Malnourished (SAM) Cases and Controls at Admission, Day 7 and Day 28 eTable 4. Comparison of Total Number of Arrhythmias and Number of Affected Severely Malnourished (SAM) Cases and Controls eTable 5. Summary on Haemodynamic Indices Supporting Figure 1 eTable 6. Comparison of Cardiac Dimensions Represented as Raw Values and z-Scores Between Children With Marasmus and Kwashiorkor at Admission, Day 7 and Day 28 eTable 7. Comparing Median Left Ventricular Mass and Left Ventricular Mass Indices Between Those With Marasmus and Kwashiorkor at Admission, Day 7 and Day 28 eTable 8. Prevalence of Pericardial Effusions Among Malnourished Children Overall and Comparison Between Children With Marasmus and Kwashiorkor at Admission, Day 7 and Day 28 eTable 9. Cardiac Function Indices in Malnourished Children Overall and Controls and Children With Marasmus and kwashiorkor at Day 7 eTable 10. Cardiac Function Indices in Malnourished Children Overall and Controls and Children With Marasmus and Kwashiorkor at Day 28 eTable 11. Bedside Observations and Laboratory Findings in Severely Malnourished Participants With Shock at Onset of Fluid Resuscitation/Rehydration eTable 12. Summary of Changes in Cardiovascular Parameters in Response to Fluid Resuscitation/Rehydration eTable 13. Causes of Death and Relationship to Intravenous Fluid Therapy for the 14 Severely Malnourished Children Who Died eTable 14. Baseline Characteristics Between Recruited and Non-Recruited Severely Malnourished Children at Admission eFigure 1. z-Scores in Cases and Controls of Cardiac Dimensions at Admission (Day 0), Day 7 and Day 28 eFigure 2. Vital Features Over Time in Cases and Controls eFigure 3. F [file jamanetwopen-2-e191054-s001.pdf]

Supplementary Online Content

Brent B, Obonyo N, Akech S, et al. Assessment of myocardial function in Kenyan children with severe, acute malnutrition: the Cardiac Physiology in Malnutrition (CAPMAL) study. *JAMA Netw Open*. 2019;2(3):e191054. doi:10.1001/jamanetworkopen.2019.1054

eMethods. Detailed Methodology

eTable 1. Day 7 Clinical and Laboratory Findings Between Severely Malnourished (SAM) Children and Controls

eTable 2. Day 28 Clinical and Laboratory Findings Between Severely Malnourished (SAM) Children and Controls

eTable 3. Comparison of Mean R/S Voltage Between Children With Severely Malnourished (SAM) Cases and Controls at Admission, Day 7 and Day 28

eTable 4. Comparison of Total Number of Arrhythmias and Number of Affected Severely Malnourished (SAM) Cases and Controls

eTable 5. Summary on Haemodynamic Indices Supporting Figure 1

eTable 6. Comparison of Cardiac Dimensions Represented as Raw Values and z-Scores Between Children With Marasmus and Kwashiorkor at Admission, Day 7 and Day 28

eTable 7. Comparing Median Left Ventricular Mass and Left Ventricular Mass Indices Between Those With Marasmus and Kwashiorkor at Admission, Day 7 and Day 28

eTable 8. Prevalence of Pericardial Effusions Among Malnourished Children Overall and Comparison Between Children With Marasmus and Kwashiorkor at Admission, Day 7 and Day 28

eTable 9: Cardiac function indices in malnourished children overall and controls and children with marasmus and kwashiorkor at day 7

eTable 10: Cardiac function indices in malnourished children overall and controls and children with marasmus and kwashiorkor at day 28

eTable 11. Bedside Observations and Laboratory Findings in Severely Malnourished Participants With Shock at Onset of Fluid Resuscitation/Rehydration

eTable 12. Summary of Changes in Cardiovascular Parameters in Response to Fluid Resuscitation/Rehydration

eTable 13. Causes of Death and Relationship to Intravenous Fluid Therapy for the 14 Severely Malnourished Children Who Died

eTable 14. Baseline Characteristics Between Recruited and Non-Recruited Severely Malnourished Children at Admission

eFigure 1. z-Scores in Cases and Controls of Cardiac Dimensions at Admission (Day 0), Day 7 and Day 28

eFigure 2. Vital Features Over Time in Cases and Controls

© 2019 Brent B et al. *JAMA Network Open.*

eFigure 3. Frank Starling Diagram of Individual Malnourished Children Pre- And Post-Fluid Resuscitation

eReferences

This supplementary material has been provided by the authors to give readers additional information about their work.

© 2019 Brent B et al. *JAMA Network Open.*

eMethods. Detailed Methodology

1. Explanation of parameters used to measure myocardial function

Fractional shortening (FS) is a measure of circumferential left ventricular shortening (load dependent)

Mitral and tricuspid annular plane systolic excursions (**MAPSE** and **TAPSE**) are measures of the longitudinal shortening of the ventricles (load-dependent parameters).

Early systolic mitral annular velocity is derived using tissue Dopplers' (load-independent measure) of longitudinal left ventricular function. The normal reference range for FS is 28-44%, the normal range for the other parameters depend on the patient´s body size.

Early to late diastolic filling ratios (E/A ratio) deliver information about the relaxation and compliance properties of the left ventricle. Reduced relaxation leads to low early diastolic mitral annular velocities (Ea). High early filling to early diastolic mitral annular velocities ratios (E/Ea ratio) are a sign of high left ventricular filling pressures. Normal reference ranges depend on the child´s BSA.

The Tei Index represents global cardiac function and has a normal reference range of 0.24 to 0.44.

2. Calculation of the Myocardial indices

Left ventricular mass (LV mass)

This was calculated using the following formula by Devereux[1] :

$$\text{LV mass (ASE)} = 0.8\ (1.04\ ((\text{LVIDd} + \text{LVPWd} + \text{IVSd}^3 - (\text{LVIDd})^3)) + 0.6\ \text{g}.$$

Systolic function

Circumferential systolic function was assessed using m-mode derived Fractional shortening (FS %). Longitudinal systolic function was assessed using mitral and tricuspid annular systolic excursions (MAPSE and TAPSE). Results were stratified using published reference values for body surface area (BSA) using the Haycock formula for BSA.[2] To calculate BSA in kwashiorkor children we used the oedema-free weight to avoid over-estimation of BSA. Tissue Doppler imaging (TDI) was used to measure the early systolic (S´) lateral mitral annular velocities using the standard technique.[3]

Systolic dysfunction was defined as reduction in FS and/or low MAPSE and/or low TAPSE and/or low S velocity outside the published normal ranges.[4-6]

Diastolic function

Pulse wave mitral inflow Doppler tracings were used to measure E/A ratio in the usual manner and published reference values were applied to define normality.[4] Early diastolic (E´) lateral mitral annular velocity was obtained using TDI. Diastolic dysfunction was defined as an abnormal E/A ratio and/or low E´ velocity and/or high E/ E´ ratio outside the published reference ranges.[4-6]

© 2019 Brent B et al. *JAMA Network Open.*

Global cardiac function

This was assessed using the Tei Index.[7] For fractional shortening and the Tei Index reference ranges of 28-44%[4] and 0.24-0.45[8] were used respectively.

3. Calculation of Haemodynamic measurements

These included cardiac index (CI), stroke volume index (SVI) and systemic vascular resistance index (SVR).

Cardiac index was calculated as

CI= (Stroke volume x heart rate)/ BSA

The latter was calculated using the following formula which does not take central venous pressure into account and therefore assumes a CVP of 0:

SVR = Mean blood pressure/VelocityTimeIntegral$_{aorta}$

Cut-offs for normality of haemodynamic parameters were derived using the data collected by Cattermole and stratified by height rather than age.[9]

The inferior vena cava collapsibility index (IVCCI) served as a surrogate marker of intravascular filling status and was calculated as

IVCCI= (IVCmax – IVCmin)/IVCmax)) x 100.

Z-score regression equations derived by Peterson et al were applied to our data for all intracardiac dimensions.[10]

4. ECG methods

Total voltage was defined as the sum of all QRS amplitudes across the 12 leads,[11] low voltage was defined as mean total limb voltage of <0.5mV or mean precordial voltage of <1mV.[12] A QRS-T angle of >90° was defined as abnormal.[13] **Heart rate variability** was defined as the difference between the maximum and minimum length (ms) of RR intervals during the first 10 consecutive RR intervals of each 12 lead ECG recording.

© 2019 Brent B et al. *JAMA Network Open.*

eTable 1. Day 7 Clinical and Laboratory Findings Between Severely Malnourished (SAM) Children and Controls

Category	Feature present at Day 7	SAM cases n=85		Controls n=18		p value
Temperature	Fever (>37.5°C)	9	(11%)	3	(17%)	0.47
	Hypothermia (< 35.0°C)	0	(0.0%)	0	(0%)	N/A
Respiratory Findings	Tachypnoea[a]	24	(2%)	7	(39%)	0.28
	Indrawing	2	(2%)	0	(0%)	0.51
	Deep breathing	6	(7%)	0	(0%)	0.25
	Hypoxia (<95%)	4	(5%)	0	(0%)	0.35
Cardiovascular Findings	Tachycardia[b]	5	(6%)	0	(0%)	0.29
	CRT >2 sec	1	(1%)	0	(0%)	0.64
	Weak pulse	1/84	(1%)	0	(0%)	0.64
	Temperature gradient[c]	3	(4%)	0	(0%)	0.42
	Hypotension[d]	6	(7%)	0	(0%)	0.25
	Gallop rhythm	2/82	(2%)	0/15	(0%)	0.54
	Hepatomegaly[e]	19	(22%)	0	(0%)	0.03
	Neck vein distension	2	(2%)	0/17	(0%)	0.52
Hydration Status	Decreased skin turgor	1/82	(1 %)	0	(0%)	0.64
	Sunken eyes	3	(4%)	1	(6%)	0.69
	Dry mucous membranes	2	(2%)	0	(0%)	0.51
	Blantyre Coma Score ≤ 4	8	(9%)	0	(0%)	0.18
Clinical Syndromes	Pneumonia (WHO)[f]	5	(6%)	0	(0%)	0.29
	Diarrhoea (>3stools/24hrs)	20	(24%)	1	(6%)	0.09
	SIRS[g]	28	(33%)	6	(33%)	0.97
Haematology	Severe anaemia (<5g/L)	1	(1%)	0/17	(0%)	0.65
	Leucocytosis[g]	17	(20%)	3	(17%)	0.75
	Thrombocytopenia (<80.000/mm^3)	4	(5%)	1	(6%)	0.88
Clinical biochemistry	Low sodium (<125 mmol/L)	0/81	(0%)	0/17	(0%)	N/A
	Low potassium (<2.5 mmol/L)	0/81	(0%)	0/17	(0%)	N/A
	Low corrected calcium (<2.1 mmol/L)	2/82	(2%)	0/17	(0%)	0.52
	Low magnesium (<0.75 mmol/L)	15/82	(18%)	0/17	(0%)	0.06
	Low phosphate (<0.8 mmol/L)	6/80	(8%)	0/17	(0%)	0.24
	Low Albumin (<34 g/L)	19	(22%)	0/18	(0%)	0.03
	High Creatinine (>6542 mg/dL)	3/81	(4%)	0/17	(0%)	0.42
	High Urea (>17.9 mg/dL)	4/82	(5%)	0/17	(0%)	0.35
Acidosis	Acidaemia (pH<7.2)	8/81	(10%)	0/17	(0%)	0.18
	Base deficit >10mmol/L	25/76	(33%)	1/16	(6%)	0.031
Cardiac markers	High Lactate (>3mmol/L)	24/77	(31%)	5/18	(28%)	0.78
	NT-pro-BNP elevation[h]	17/43	(40%)	2/10	(20)	0.25
Cardiac Mass (g)	Median ventricular mass (LVM)	26	[20,36]	33	[28,49]	0.006
	Median LVM indexed to height$^{2.7}$	68	[58,80]	70	[54,88]	0.66
	Median LVM indexed to BSA	58	47,70]	61	[51,74]	0.78

Data presented as absolute numbers and proportions (%) and medians and interquartile range [IQR].

Legend: See Below eTable 2

© 2019 Brent B et al. *JAMA Network Open.*

eTable 2. Day 28 Clinical and Laboratory Findings Between Severely Malnourished (SAM) Children and Controls

Category	Feature present at Day 28	SAM cases n=62		Controls n=19		p value
Temperature	Fever (>37.5°C)	5	(8 %)	1	(5%)	0.69
	Hypothermia (< 35.0°C)	0	(0%)	0	(0%)	N/A
Respiratory Findings	Tachypnoea[a]	12	(19%)	5/17	(30%)	0.37
	Indrawing	1	(2%)	0	(0%)	0.62
	Deep breathing	0	(0%)	0	(0%)	0.12
	Hypoxia (<95%)	2	(4%)	0	(0%)	0.54
Cardiovascular Findings	Tachycardia (EPLS)[b]	2	(4%)	0	(0%)	0.43
	Bradycardia (<80/min)	0	(0%)	0	(0%)	N/A
	CRT >2 sec	0	(0%)	0	(0%)	N/A
	Weak pulse	0	(0%)	0	(0%)	N/A
	Temperature gradient[c]	0	(0%)	0	(0%)	N/A
	Hypotension[d]	2	(3%)	0	(0%)	0.43
	Gallop rhythm	1/61	(2%)	0/18	(0%)	0.59
	Hepatomegaly[e]	13	(21%)	1	(5%)	0.11
	Neck vein distension	1/61	(2%)	0	(0%)	0.58
Hydration Status	Decreased skin turgor	0/61	(0%)	0/18	(0%)	N/A
	Sunken eyes	0	(0%)	0	(0%)	N/A
	Dry mucous membranes	0/61	(0%)	0	(0%)	N/A
	Blantyre Coma Score ≤ 4	3	(5%)	0	(0%)	0.33
Clinical Syndromes	LRTI (WHO)[f]	2	(3%)	2	(11%)	0.22
	Diarrhoea (>3stools/24hrs)	1	(2%)	0	(0%)	0.58
	SIRS[g]	16	(26%)	7	(36%)	0.35
Haematology	Severe anaemia (<5g/dL)	1	(1%)	0	(0%)	0.58
	Leucocytosis[g]	4	(7%)	4	(21%)	0.062
	Thrombocytopenia (<80.000/mm^3)	1	(2%)	2	(11%)	0.072
Clinical biochemistry	Low sodium (<125 mmol/L)	0/57	(0%)	0/17	(0%)	N/A
	Low potassium (<2.5 mmol/L)	0/57	(0%)	0/17	(0%)	N/A
	Low corrected calcium (<8.4 mg/dL)	0/57	(0%)	0/17	(0%)	N/A
	Low magnesium (<1.5 mEq/L)	2/57	(4%)	0/16	(0%)	0.45
	Low phosphate (<2.5 ng/mL)	0/57	(0%)	0/17	(0%)	N/A
	Low Albumin (<34 g/L)	0/62	(0%)	0	(0%)	N/A
	High Creatinine (>6542 mg/dL)	1/57	(2%)	0/17	(0%)	0.58
	High Urea (>17.9 mg/dL)	2/57	(4%)	0/17	(0%)	0.43
Acidosis	Acidaemia (pH<7.2)	1/56	(2%)	1/15	(7%)	0.31
	Base deficit >10mmol/L	5/56	(9%)	1/13	(8%)	0.89
Cardiac markers	High Lactate (>3mmol/L)	8/54	(15%)	6/17	(35%)	0.06
	NT-pro-BNP elevation[h]	9/23	(39%)	4/9	(44%)	0.78

Data presented as absolute numbers and proportions (%) and medians and IQR**.**

© 2019 Brent B et al. *JAMA Network Open.*

Footnotes for Table e1 and eTable 2

[a] Tachypnoea was defined as respiratory rate (RR)>= 50/min in children aged 2-11months, RR>=40/min in those aged 1-5 years and RR >=30 in children >5 years as per WHO definition[14]

[b] EPLS tachycardia was defined as a HR>180/min in a child <12months and a HR>160/min in children aged 1-5 years

[c] The temperature gradient (TG) was assessed by running the back of the hand from the toe to the knee. A positive gradient was defined as a temperature change from cold to warm.

[d] Paediatric Advanced Life Support (PALS) hypotension was defined as a systolic BP <70mmHg in children <12months, <70mmHg+(2*age in years (y)) in those aged 1-10y and <90mmHg in children >10y[15]

[e]Hepatomegaly was defined as a palpable liver edge ≥ 2cm below the costal margin in the mid-clavicular line.

[f] The term pneumonia includes mild, moderate and severe forms as defined by WHO.

[g]SIRS and leucocytosis were defined using the criteria by Goldstein et al[16]

[h] Elevated NT-proBNP was defined by Nir[17] as >650pg/ml in children up to 12m of age, >400pg/ml in children aged 12m-<24mth, >300 in children aged 2y-<6y and >160pg/ml for children >6y

© 2019 Brent B et al. *JAMA Network Open.*

eTable 3. Comparison of Mean R/S Voltage Between Children With Severely Malnourished (SAM) Cases and Controls at Admission, Day 7 and Day 28

Lead	Day 0					Day 7					Day 28				
	SAM cases N=88		Controls N=22		p value	SAM cases N=83		Controls N=18		p value	SAM cases N=61		Controls N=19		p value
	Mean	SD	Mean	SD		Mean	SD	Mean	SD		Mean	SD	Mean	SD	
I (µV)	792	452	1035	351	0.02	886	427	1056	393	0.12	964	381	972	393	0.94
II (µV)	947	493	1138	283	0.09	855	363	1067	234	0.02	929	369	1062	239	0.14
III (µV)	645	402	756	480	0.27	588	347	657	424	0.46	676	583	670	410	0.96
aVR (µV)	322	318	459	377	0.09	374	410	564	430	0.08	349	367	524	446	0.09
aVL (µV)	606	391	853	400	0.01	684	402	833	425	0.16	765	430	758	380	0.95
aVF (µV)	714	444	814	430	0.35	580	352	738	389	0.09	692	359	736	338	0.63
V1 (µV)	1491	757	1771	623	0.11	1565	761	1984	842	0.04	1484	577	1869	570	0.01
V2 (µV)	2886	1039	3453	749	0.02	2999	1052	3670	870	0.01	3066	911	3813	1010	0.003
V3 (µV)	3141	1186	3733	932	0.03	2827	1027	3651	1054	0.003	2881	848	3695	1055	<0.001
V4 (µV)	3133	1275	4207	1080	<0.001	2744	1078	3783	1198	<0.001	2933	927	4030	1110	<0.001
V5 (µV)	2010	959	3129	1482	<0.001	1869	780	2772	1092	<0.001	2070	822	3019	961	<0.001
V6 (µV)	1109	559	2102	1112	<0.001	1063	453	1712	770	<0.001	1293	549	1812	494	<0.001
Total (µV)	1483	496	1954	418	<0.001	1419	400	1874	415	<0.001	1508	323	1913	351	<0.001
Total limb (µV)	671	299	842	197	0.01	661	258	819	202	0.02	729	257	787	184	0.36

Data presented are mean microvolts and standard deviations (SD)

© 2019 Brent B et al. *JAMA Network Open.*

eTable 4. Comparison of Total Number of Arrhythmias and Number of Affected Severely Malnourished (SAM) Cases and Controls

		**SAM cases** N=55		**Controls** N=18		p value
	No. children	3	(6%)	0	(0%)	0.31
Bradycardia	Total no. episodes	13		0		-
	No. episodes/day*	0.06		0		0.41
	No. children	22	(40%)	4	(22%)	0.17
SVT	Total no. episodes	392		19		-
	No. episodes/day*	1.4		0.3		0.37
	No. children	13	(24%)	3	(17%)	0.54
Bigemini	Total no. episodes	254		7		-
	No. episodes/day*	0.8		0.1		0.28
	No. children	13	(24%)	3	(17%)	0.56
Trigemini	Total no. episodes	5		6		-
	No. episodes/day*	0.02		0.1		0.07
	No. children	33	(60%)	8	(44%)	0.25
Premature	Total no. episodes	1353		746		-
Ventricular contraction	No. episodes/day*	5		12		0.13
	No. children	23	(42%)	4	(22%)	0.14
Couplet	Total no. episodes	2133		413		-
	No. episodes/day*	9		7		0.72
	No. children	10	(18%)	2	(11%)	0.48
Triplet	Total no. episodes	387		44		-
	No. episodes/day*	2		1		0.51
	No. children	10	(18%)	3	(17%)	0.88
Salvos	Total no. episodes	385		10		-
	No. episodes/day*	2		0.2		0.33
	No. children	30	(55%)	6	(33%)	0.12
R on T	Total no. episodes	3086		943		-
	No. episodes/day*	14		15		0.92
	No. children	7	(13%)	1	(6%)	0.40
Ventricular	Total no. episodes	50		1		-
Tachycardia	No. episodes/day*	0.2		0.02		0.447
Significant ventricular arrhythmias	No. children	33	(60%)	6	(33%)	0.049
	Total no. episodes	6041		1411		
	No. episodes/day*	26		22		0.79

* Mean number of episodes per 24-hour period of Holter monitoring

eTable 5. Summary on Haemodynamic Indices Supporting Figure 1

	**Prior to fluid**		**Post-rehydration**						
Timing (Hours)	0 hour		1 hour		8 hours		24 hours		χ^2 trend
Low Fractional Shortening	3/13	(23%)	2/12	(17%)	0/6	(0%)	1/11	(9%)	0.60
Low MAPSE	1/10	(10%)	0/8	(0%)	1/5	(17%)	2/10	(20%)	0.41
Low TAPSE	3/10	(30%)	2/8	(25%)	2/7	(29%)	2/11	(18%)	0.89
High E/E’	1/8	(13%)	1/8	(13%)	1/3	(25%)	0/9	(0%)	0.56
Low Cardiac Index	4/13	(31%)	3/12	(25%)	2/6	(33%)	0/11	(0%)	0.36
High SVRI	9/11	(82%)	7/12	(58%)	4/7	(57%)	5/9	(56%)	0.69

© 2019 Brent B et al. *JAMA Network Open.*

eTable 6. Comparison of Cardiac Dimensions Represented as Raw Values and z-Scores Between Children With Marasmus and Kwashiorkor at Admission, Day 7 and Day 28

		Admission					Day 7					Day 28				
Dimension		Marasmus		Kwashiorkor		p value	Marasmus		Kwashiorkor		p value	Marasmus		Kwashiorkor		p value
		N=48			N=35			N=48		N=34			N=38		N=24	
Systolic	IVSs (mm)	8	(7, 9)	8	(7, 9)	0.93	8	(7, 9)	8	(7, 9)	0.85	8	(7, 9)	8	(8, 9)	0.46
	z-IVSs	*1.8*	*(1.0, 2.1)*	*1.4*	*(0.9, 2.0)*	*0.20*	*1.8*	*(1.0, 2.1)*	*1.7*	*(0.9, 2.0)*	*0.38*	*1.6*	*(1.2, 2.5)*	*1.6*	*(1.5, 2.1)*	*0.96*
	LVIDs (mm)	15	(13, 17)	15	(14, 17)	0.69	16	(14, 19)	15	(14, 17)	0.21	16	(15, 19)	16	(15, 18)	0.90
	z-LVIDs	*-0.02*	*(-0.6, 1.0)*	*-0.02*	*(-0.8, 0.6)*	*0.33*	*0.5*	*(-0.5, 1.5)*	*-0.5*	*(-1.1, 0.2)*	*0.003*	*0.3*	*(-0.6, 1.0)*	*0.1*	*(-0.7, 0.8)*	*0.42*
	LVPWs (mm)	7	(6, 8)	7	(6, 7)	0.40	7	(6, 8)	7	(6, 8)	0.70	8	(6, 9)	7	(7, 8)	0. 86
	z-LVPWs	*0.1*	*(-0.7, 0.9)*	*-0.6*	*(-0.9, 0.2)*	*0.02*	*0.3*	*(-0.3, 0.8)*	*0.2*	*(-0.7, 0.3)*	*0.19*	*0.7*	*(-0.5, 1.3)*	*0.3*	*(-0.2, 0.8)*	*0.44*
Diastolic	IVSd (mm)	6	(5, 7)	6	(5, 7)	0.77	6	(6, 7)	6	(6, 7)	0.62	7	(6, 8)	7	(7, 7)	0.34
	z-IVSd	*1.6*	*(1.0, 2.3)*	*1.6*	*(0.8, 1.9)*	*0.27*	*1.9*	*(1.1, 2.4)*	*1.7*	*(1.3, 2.4)*	*0.78*	*1.9*	*(1.5, 2.5)*	*2.2*	*(1.7, 2.4)*	*0.53*
	LVIDd (mm)	24	(22, 27)	25	(22, 27)	0.62	25	(23, 29)	24	(23, 26)	0.52	26	(23, 29)	26	(24, 28)	0.96
	z-LVIDd	*-0.1*	*(-1.0, 0.8)*	*-0.3*	*(-1.3, 0.4)*	*0.22*	*0.08*	*(-0.5, 1.0)*	*-0.5*	*(-1.3, 0.2)*	*0.002*	*0*	*(-0.5, 1.0)*	*0.2*	*(-0.8, 0.7)*	*0.55*
	LVPWd (mm)	4	(4, 5)	4	(4, 5)	0.92	5	(4, 5)	5	(4, 5)	0.93	5	(4, 5)	5	(4, 6)	0.53
	z-LVPWd	*1.1*	*(0.7,1.8)*	*0.9*	*(0.5, 1.7)*	*0.26*	*1.2*	*(0.8, 2.0)*	*1*	*(0.6,1.9)*	*0.27*	*1.5*	*(0.9, 2.0)*	*1.6*	*(0.7, 2.5)*	*0.82*
	LV dilatation	3	-6%	1	-3%	0.48	6	-13%	0	0%	0.03	4	-11%	0	0%	0.09

Data is presented as medians (IQR), z-scores are presented in italic font.
The following abbreviations were used:
IVSs=intraventricular septum in systole, IVSd=intraventricular septum in diastole, LVIDs= left ventricular inner diameter in systole, LVIDd= left ventricular inner diameter in diastole, LVPWs=left ventricular posterior wall in systole, LVPWd=left ventricular posterior wall in diastole.

© 2019 Brent B et al. *JAMA Network Open.*

eTable 7. Comparing Median Left Ventricular Mass and Left Ventricular Mass Indices Between Those With Marasmus and Kwashiorkor at Admission, Day 7 and Day 28

	All SAM		Marasmus		Kwashiorkor		p value
**Admission**	N=83		N=48		N=35		
LVM (g)	23	(17, 31)	23	(17, 31)	23	(16, 29)	0.86
$LVMI_1$ (g/m^2)	63	(51, 77)	65	(53, 78)	56	(46, 72)	0.14
$LVMI_2$ ($g/m^{2.7}$)	50	(42, 62)	53	(45, 66)	45	(38, 57)	0.01
**Day 7**	N=82		N=48		N=34		
LVM (g)	26	(20, 36)	26	(20, 36)	25	(20, 35)	0.71
$LVMI_1$ (g/m^2)	68	(58, 80)	74	(63, 86)	65	(54, 75)	0.02
$LVMI_2$ ($g/m^{2.7}$)	58	(47, 70)	65	(55, 75)	52	(46, 62)	0.004
**Day 28**	N=59		N=35		N=24		
LVM (g)	31	(23, 39)	34	(23, 39)	30	(26, 38)	0.73
$LVMI_1$ (g/m^2)	77	(65, 92)	80	(64, 92)	75	(65, 91)	0.90
$LVMI_2$ ($g/m^{2.7}$)	67	(58, 81)	73	(59, 85)	61	(56, 74)	0.15

Data are presented as medians (IQR)

Three methods to describe Left ventricular mass (LVM) presented as raw data (LVM), indexed to height ($LVMI_1$) and indexed to BSA ($LVMI_2$) as recommended by Haycock *el al*.[2]

© 2019 Brent B et al. *JAMA Network Open.*

eTable 8. Prevalence of Pericardial Effusions Among Malnourished Children Overall and Comparison Between Children With Marasmus and Kwashiorkor at Admission, Day 7 and Day 28

**Time point**	**Overall n= 88**		**Marasmus n=52**		**Kwashiorkor n=36**		**P value***
**Admission**							
Small effusion (<0.5 cm)	18	(20%)	7	(14%)	11	(31%)	-
Large effusion (>0.5 cm)	2	(2%)	1	(2%)	1	(3%)	-
Total	20	(23%)	8	(15%)	12	(33%)	0.05
**Day 7**							
Small effusion (<0.5 cm)	14	(17%)	8	(16%)	6	(18%)	-
Large effusion (>0.5 cm)	6	(7%)	5	(10%)	1	(3%)	--
Total	20/85	(24%)	13/51	(26%)	7/34	(21%)	0.60
**Day 28**							
Small effusion (<0.5 cm)	11	(18%)	5	(14%)	6	(24%)	-
Large effusion (>0.5 cm)	2	(3 %)	2	(5%)	0	(0%)	-
Total	13/62	(21%)	7/37	(19%)	6/25	(24%)	0.63

*p value comparing marasmus and kwashiorkor
Data is presented as absolute numbers and proportions (%)

© 2019 Brent B et al. *JAMA Network Open.*

eTable 9: Cardiac function indices in malnourished children overall and controls and children with marasmus and kwashiorkor at day 7

**Day 7**		Malnourished N=82		Controls N=18		p value	Marasmus N=48		Kwashiorkor N=34		p value
Systolic	FS (%)	37	(34-41)	36.5	(34-43)	0.53	36	(34-40)	38	(35-42)	0.21
	EF (%)	69	(64-74)	69	(65-75)	0.72	68.5	(64-73)	70.5	(66-74)	0.19
	MAPSE	9	(8-10)	10	(9-11)	0.12	9	(8-10)	9	(9-10)	0.55
	TAPSE	14.5	(13-17)	18.5	(15-21)	0.001	14	(13-16)	15	(13-17)	0.22
	S´ (cm/s)	5	(5-6)	7	(6-8)	0.002	5	(4-6)	0.06	(5-6)	0.26
Diastolic	E/A ratio	1.26	(1.1-1.4)	1.31	(1.2-1.4)	0.15	1.29	(1.1-1.5)	1.18	(1.09-1.33)	0.08
	E´ (cm/s)	11	(9-12)	12	(10-15)	0.03	10	(9-12)	0.11	(9-12)	0.82
	E/E' (m/s)	7.7	(6.4-9.3)	7.9	(6.2-8.49)	0.72	8.3	(6.9-9.6)	6.78	(5.85-8.29)	0.038
Global	Tei Index	0.31	(0.24-0.42)	0.33	(0.25-0.44)	0.49	0.33	(0.26-0.46)	0.28	(0.19-0.37)	0.039
Haemodynamic	SV (ml)	15	(12, 19)	25	(21, 30)	<0.001	14	(12, 21)	15	(12, 16)	0.85
	SVI (ml/m2)	39	(31, 46)	49	(41, 54)	0.007	40	(32, 49)	38	(30, 42)	0.10
	CO (l/min)	1.97	(1.67, 2.63)	2.82	(2.38, 3.65)	0.001	2.05	(1.63, 2.65)	1.9	(1.72, 2.39)	0.73
	CI (l/min/m2)	5.21	(4.46, 6.32)	5.50	(4.69, 6.83)	0.46	5.27	(4.53, 7.04)	5.02	(4.22, 5.88)	0.08
	SVR (dyn·s/cm^5)	442	(365, 513)	355	(299, 451)	0.004	442	(366, 513)	446	(356, 520)	0.97
	SVRI dyn s/cm ^{5}/m^2	1093	(932, 1472)	703	(510, 836)	<0.001	1112	(953, 1523)	1033	(881, 1331)	0.13

Abbreviations: CI, cardiac index; CO, cardiac output; Ea, early diastolic mitral annular velocities; E/E', ratio of mitral peak velocity of early filling to early diastolic mitral annular velocity; EF, ejection fraction; FS, fractional shortening; IQR, interquartile range; MAPSE, mitral annular plane systolic excursion; NA, not applicable; SAM, severe, acute malnutrition; SV, stroke volume, SVI, SV index; SVR, systemic vascular resistance; SVRI, SVR index; TAPSE, tricuspid annular plane systolic excursion.

© 2019 Brent B et al. *JAMA Network Open.*

eTable 10: Cardiac function indices in malnourished children overall and controls and children with marasmus and kwashiorkor at day 28

**Day 28**		Malnourished N=59		Controls N=19		p value	Marasmus N=35		Kwashiorkor N=24		p value
Systolic	FS (%)	37	(33, 39)	35	(33, 39)	0.89	36	(32, 39)	37	(34, 39)	0.55
	MAPSE	9	(8, 10)	10	(8, 12)	0.08	9	(8, 10)	10	(8, 10)	0.09
	TAPSE	16	(14, 18)	17	(14, 20)	0.26	16	(14, 18)	16	(13, 19)	0.87
	S´(cm/s)	6	(5, 7)	7	(5, 9)	0.015	6	(5, 7)	6	(5, 7)	0.35
Diastolic	E/A ratio	1.22	(1.11, 1.38)	1.37	(1.31, 1.55)	0.002	1.24	(1.07, 1.37)	1.2	(1.15, 1.41)	0.77
	E (cm/s)	11	(9, 13)	13	(10, 15)	0.06	11	(9, 15)	11	(10, 12)	0.95
	E/E' (m/s)	7.9	(6.7, 10.1)	7.5	(6.1, 8.2)	0.37	8.4	(6.6, 10.4)	7.5	(6.8, 9.0)	0.57
Global	Tei Index	0.29	(0.23, 0.42)	0.35	(0.31, 0.43)	0.21	0.3	(0.26, 0.43)	0.28	(0.19, 0.42)	0.19
Haemodynamic	SV (ml)	16	(13, 21)	22	(17, 25)	0.011	16	(12, 21)	16	(13, 21)	0.88
	SVI (ml/m2)	41	(34, 50)	42	(35, 48)	0.81	42	(33, 51)	41	(34, 48)	0.62
	CO (l/min)	2.55	(1.7, 2.87)	2.55	(1.93, 3.05)	0.47	2.45	(1.68, 3.13)	2.29	(1.81, 2.79)	0.84
	CI (l/min/m2)	5.96	(4.43, 6.84)	5.07	(3.86, 5.71)	0.013	6.14	(4.43, 7.13)	5.77	(4.40, 6.41)	0.37
	SVR (dyn·s/cm^5)	425	(370, 540)	334	(275, 442)	0.003	442	(384, 563)	414	(369, 472)	0.29
	SVRI dyn s/cm ^{5}/m^2	1044	(926, 1443)	674	(495, 944)	<0.001	1137	(993, 1491)	1014	(868, 1211)	0.11

Abbreviations: CI, cardiac index; CO, cardiac output; Ea, early diastolic mitral annular velocities; E/E', ratio of mitral peak velocity of early filling to early diastolic mitral annular velocity; EF, ejection fraction; FS, fractional shortening; IQR, interquartile range; MAPSE, mitral annular plane systolic excursion; NA, not applicable; SAM, severe, acute malnutrition; SV, stroke volume, SVI, SV index; SVR, systemic vascular resistance; SVRI, SVR index; TAPSE, tricuspid annular plane systolic excursion.

© 2019 Brent B et al. *JAMA Network Open.*

eTable 11. Bedside Observations and Laboratory Findings in Severely Malnourished Participants With Shock at Onset of Fluid Resuscitation/Rehydration

**Category**	**Features at time of shock**	**Episodes of shock =15**	
Temperature	Fever (>37.5°C)	3/12	(25%)
	Hypothermia (< 35.0°C)	1/13	(8%)
Respiratory	Tachypnoea[a]	10/14	(71%)
	Deep breathing	13/13	(100%)
	Hypoxia (<95%)	1/14	(7%)
Cardio-vascular	Tachycardia[b]	3/14	(21%)
	CRT >3 sec	3/14	(21%)
	Weak pulse	6/13	(46%)
	Temperature gradient[c]	12/15	(80%)
	Gallop rhythm	0/12	(0%)
	Hepatomegaly[d]	3/15	(20%)
Neurological	Blantyre Coma Score ≤ 4	10/14	(71%)
Laboratory	Severe hypoalbuminaemia (<15g/L)	2/15	(13%)
	Severe hyponatraemia (<125mmol/L)	3/10	(30%)
	Severe hypokalaemia (<2.5mmol/L)	2/10	(20%)
	Lactate>3 (mmol/L)	3/7	(43%)
	Severe acidaemia (pH<7.20)	6/13	(46%)
	Base deficit >10mmol/L	11/13	(85%)
	Elevated NT proBNP[h]	4/5	(80%)

Data is presented as total numbers (%)

[a] Tachypnoea was defined as respiratory rate (RR)>= 50/min in children aged 2-11months, RR>=40/min in those aged 1-5 years and RR >=30 in children >5 years according to the WHO definitions[14]

[b] Tachycardia was defined as a HR>180/min in a child <12months and a HR>160/min in children aged 1-5 years

[c] Assessed by running the back of the hand from the toe to the knee. A positive gradient was defined as a temperature change from cold to warm.

[d] Defined as a palpable liver edge >2cm below the costal margin in the mid-clavicular line[14]

[e] Elevated NT-proBNP was defined by Nir[17]as >650pg/ml in children up to 12m of age, >400pg/ml in children aged 12m to <24m, >300 in children aged 2yto <6y and >160pg/ml for children >6 y

© 2019 Brent B et al. *JAMA Network Open.*

eTable 12. Summary of Changes in Cardiovascular Parameters in Response to Fluid Resuscitation/Rehydration

Patient	HR	SVI	CI	FS	LVIDD	SVR	Comments
C5	↓	↑	↔	↔	↑	↓	Appropriate response to fluids.
C8	↓	↑	(↓)	↑	(↓)	↓	Clinically septic with clinical signs of CCF. Appropriate response to fluids.
C22	↓	↓	(↓)	(↓)	↓	↓	Initially hyperdynamic circulation with abnormally high FS and CI. Appropriate response to fluids.
C24	↓	↑	↑	↑	↑	↓	Appropriate response to fluids.
C36	↓	↑	↑	↑	↔	↓	Appropriate response to fluids.
C37	↔	↑↑	↑	↑	↑	↓	Severe hypovolaemia and very low LVIDD initially. Appropriate physiological response to fluids, although child died within 24 hours of fluid resuscitation.
C40	↔	↑	↑	↔	↑	↓	Overall appropriate response to fluids.
C47	↓	↑	↑	↓	↑	↓	HIV infected child with sepsis and severe hypovolaemia. Appropriate physiologic response to fluids but myocardial contractility impaired and child died within 24 hours despite fluid resuscitation.
C51	↑ then ↓	↓	↓	↓ then ↑	↓ then ↑	↓	Presented in warm septic shock with myocardial dysfunction. Initial worsening of physiological parameters despite fluids but LVIDD not increased and FS recovered with further fluids so initial deterioration probably due to sepsis
C70	(↓)	↑	↑	↑	↑	-	Appropriate physiological response to fluids, although child died within 24 hours of fluid resuscitation.
C89 (a)	↑	↑	↑	↔	↑↑↑	-	HIV infected, recently started ARVs. Presented severely hypovolaemic with a hypercontractile cardiac state. Appropriate cardiovascular physiological response to IV fluid resuscitation and rehydration.
C89 (b)	↓	↓	↓	↓	↓	↑	Second episode of shock on day 5 of admission. Better intravascular filling than at admission, but has not responded to nutritional rehabilitation and developed neutropenic sepsis.
C89 (c)	↔	↑	↑	↑	↑	↓	Ongoing diarrhoea and suspected sepsis with high IL-6 (2978 pg/mL). Clinically hypovolaemic. Appropriate physiological response to fluids.
C90	↓	↑	↑	↑	↑	↑	Overall appropriate response to fluids. Reason for increase in SVRI unclear.

© 2019 Brent B et al. *JAMA Network Open.*

eTable 13. Causes of Death and Relationship to Intravenous Fluid Therapy for the 14 Severely Malnourished Children Who Died

Study No.	Malnutrition syndrome	Received IV fluids for shock	Died within 24 h of IV fluids	Death expected or unexpected	Cause of death
C1	Marasmus	No	N/A	Unexpected	Liver failure, diarrhoea, metabolic derangement
C21	Marasmus	No	N/A	Expected	Respiratory deterioration – confirmed TB
C31	Kwashiorkor	No	N/A	Unexpected	Hypovolaemic shock secondary to diarrhoea
C63	Kwashiorkor	No	N/A	Unexpected	Overwhelming sepsis, underlying cerebral palsy
C74	Kwashiorkor	No	N/A	Unexpected	Presumed sepsis, worsening abdominal distension with sudden drop in Hb
C75	Kwashiorkor	No	N/A	Unexpected	Sudden respiratory deterioration – spontaneous pneumothorax
C5	Marasmus	Yes	No	Expected	Intra-abdominal pathology with worsening abdominal distension
C24	Marasmus	Yes	No	Expected	Unable to establish enteral feeds, hypovolaemic shock, HIV
C37	Marasmus	Yes	Yes	Expected	Unable to establish enteral feeds, hypovolaemia, HIV
C47	Marasmus	Yes	Yes	Expected	Hypovolaemic shock, HIV
C89	Marasmus	Yes	No	Expected	Hypovolaemic shock secondary to diarrhoea, underlying HIV, presumed TB
C90	Marasmus	Yes	No	Expected	Hypovolaemic shock secondary to diarrhoea, underlying HIV
C55	Kwashiorkor	Yes	No	Expected	Overwhelming sepsis, hypovolaemic shock
C70	Kwashiorkor	Yes	No	Expected	Hypovolaemic shock secondary to diarrhoea, underlying cerebral palsy

© 2019 Brent B et al. *JAMA Network Open.*

eTable 14. Baseline Characteristics Between Recruited and Non-Recruited Severely Malnourished Children at Admission

**Category**	**Features at admission**	**Recruited N=88**		**Non-recruited N=230**		**p value**
Gender	Male (%)	48	(55%)	134	(58%)	0.55
Age	Median age (months)	19	(13, 35)	19	(12, 38)	0.81
Anthropometry	Median weight (kg)	6.79	(5.80, 7.96)	7.26	(6.06, 9.22)	0.007
	Median WH z-score[a]	-3.2	(-3.8, -2.5)	-3.1	(-3.6, -2.5)	0.18
	Median MUAC[b] (cm)	10.9	(10.0-11.5)	11.5	(11.0-13)	**<0.001**
Malnutrition type	Kwashiorkor	36	(41%)	70	(31%)	0.09
Temperature	Fever (>37.5°C)	25	(28%)	107	(47%)	0.24
	Hypothermia (< 35.0°C)	2	(3%)	1	(0.4%)	0.13
Respiratory	Tachypnoea (>40/min)	25	(23%)	60	(31%)	0.15
Cardiovascular	Tachycardia (>160/min)	22	(25%)	52	(23%)	0.74
	Bradycardia (<80/min)	0	(0.0%)	0	(0.0%)	N/A
Neurological	Impaired consciousness *	7	(8%)	27	(12%)	0.33
	History of convulsions	5	(6%)	17	(8%)	0.58
Clinical diagnosis	Diarrhoea *	42	(46.7%)	112	(49%)	0.69
	Pneumonia *	16	(18%)	50	(22%)	0.48
Outcome	Death (any time)	14	(16%)	25	(11%)	0.17
	Death< 48 hrs	1	(1%)	6	(3%)	0.82
	Death ≥2 days < 7days	2	(2%)	7	(3%)	0.31
	Late deaths ≥ 7 days	11	(79%)	12	(48%)	0.06

** as diagnosed by the admitting clinician*

Data is presented as absolute numbers and proportions (%) and medians and interquartile ranges (IQR).
[a]WHZ= weight-for-height z-score
[b]MUAC= Mid upper arm circumference

© 2019 Brent B et al. *JAMA Network Open.*

eFigure 1. z-Scores in Cases and Controls of Cardiac Dimensions at Admission (Day 0), Day 7 and Day 28

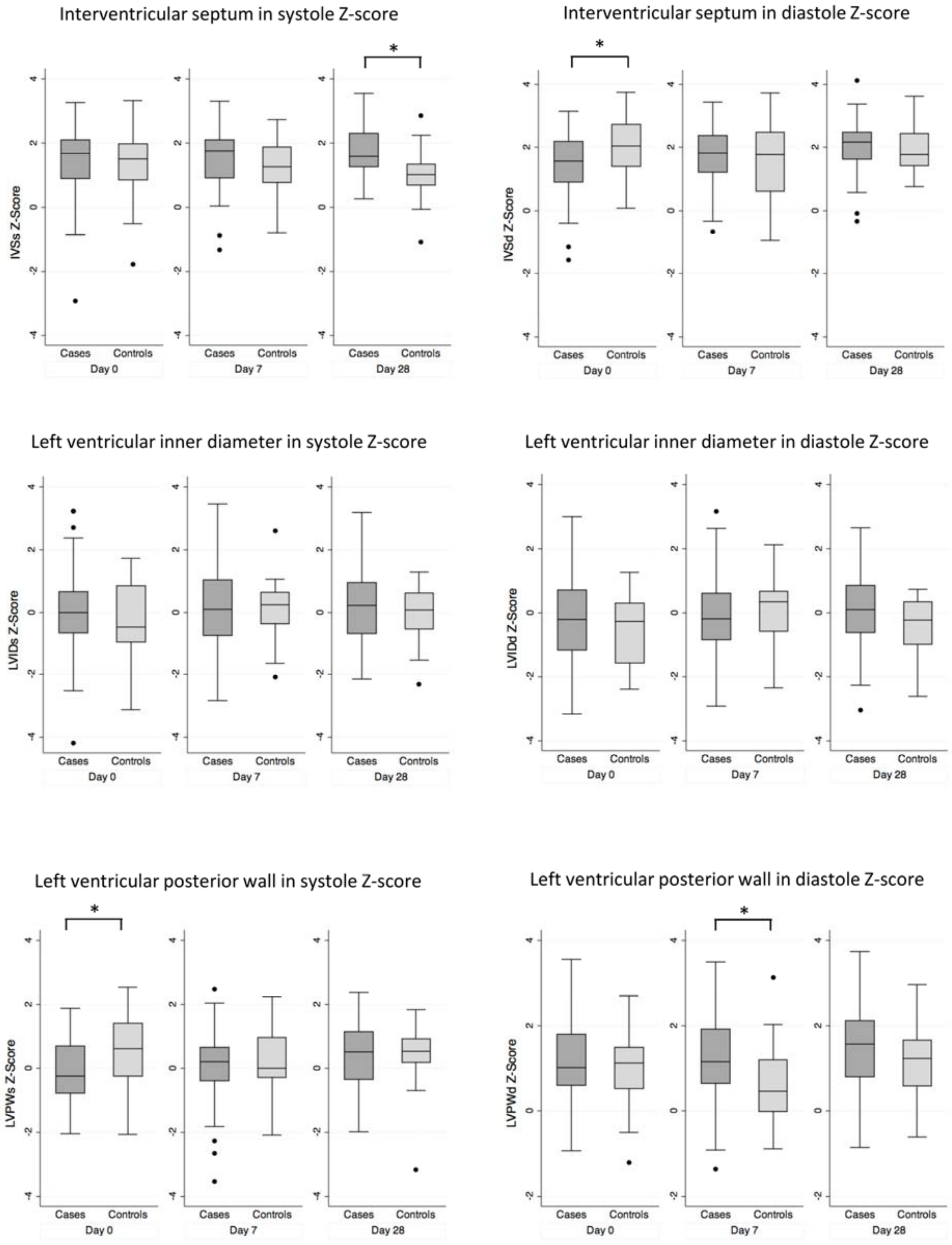

*** p< 0.05 ** P< 0.01**

© 2019 Brent B et al. *JAMA Network Open.*

eFigure 2. Vital Features Over Time in Cases and Controls

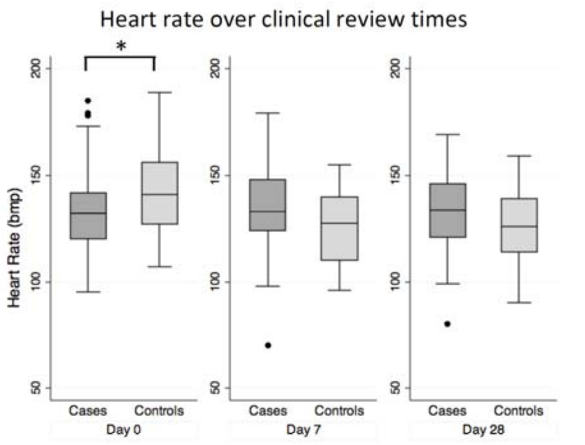

No	88	22	85	18	62	19
Median	132	141	133	128	134	126
(IQR)	(120,142)	(127, 156)	(124,148)	(110,140)	(121,146)	(114,139)

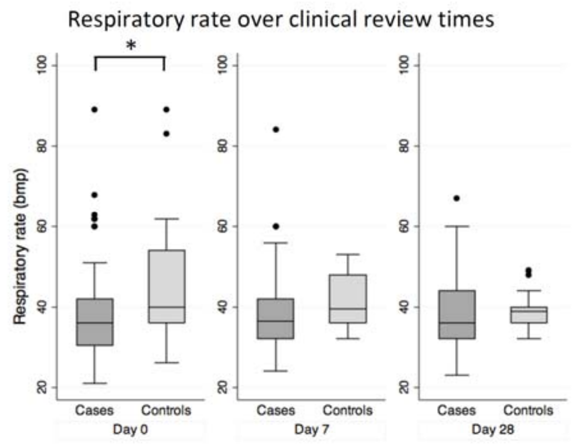

No	88	22	84	18	62	19
Median	36	40	37	40	36	39
(IQR)	(31, 42)	(36, 54)	(32, 42)	(36, 48)	(32, 44)	(36, 40)

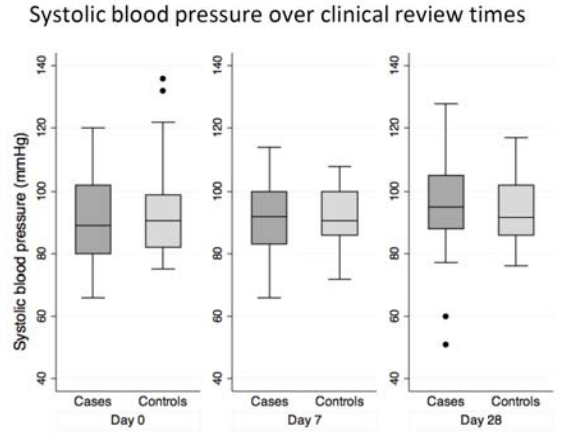

No	87	22	83	18	62	18
Median	89	91	92	91	95	92
(IQR)	(80, 102)	(82, 99)	(83, 100)	(86, 100)	(88, 105)	(86, 102)

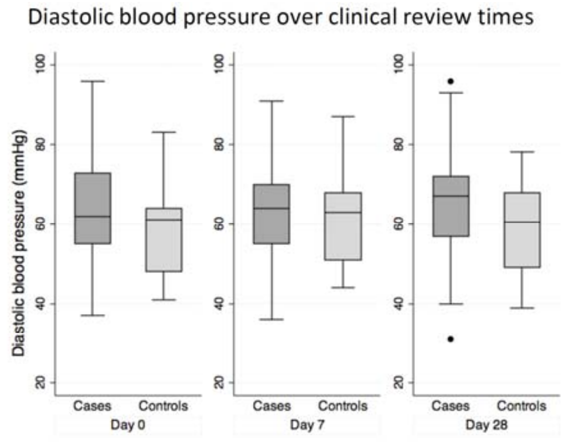

No	87	21	83	18	62	18
Median	62	61	64	63	67	61
(IQR)	(55, 73)	(48, 64)	(55, 70)	(51, 68)	(57, 72)	(49, 68)

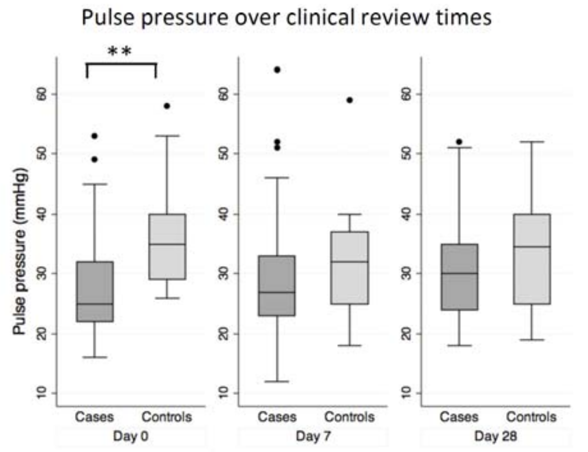

No	87	21	83	18	62	18
Median	25	35	27	32	30	35
(IQR)	(22, 32)	(29, 40)	(23, 33)	(25, 37)	(24, 35)	(25, 40)

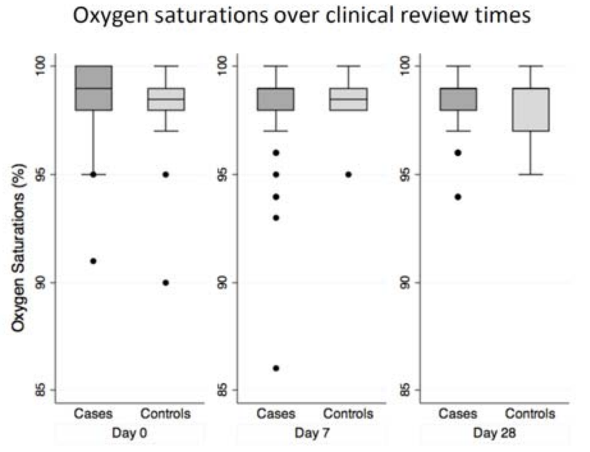

No	88	22	85	18	62	19
Median	99	99	99	99	99	99
(IQR)	(98, 100)	(98, 99)	(98, 99)	(98, 99)	(98, 99)	(97, 99)

*** p< 0.05 ****

© 2019 Brent B et al. *JAMA Network Open.*

eFigure 3. Frank Starling Diagram of Individual Malnourished Children Pre- And Post-Fluid Resuscitation

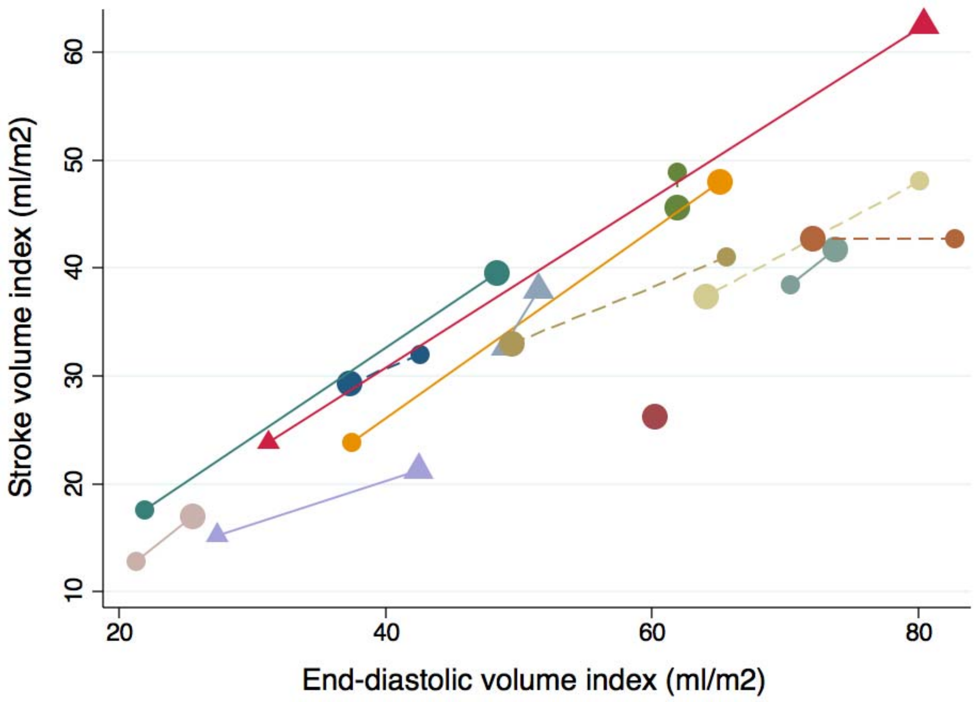

Dashed lines indicate patients with decreasing stroke volume index post fluid resuscitation
Solid lines indicate patients with improving stroke volume index
Triangles indicate patients who died within 24 hours post fluid resuscitation

© 2019 Brent B et al. *JAMA Network Open.*

eReferences

1. Devereux RB, Reichek N. Echocardiographic determination of left ventricular mass in man. Anatomic validation of the method. *Circulation.* 1977;55(4):613-618.
2. Haycock GB, Schwartz GJ, Wisotsky DH. Geometric method for measuring body surface area: a height-weight formula validated in infants, children, and adults. *The Journal of pediatrics.* 1978;93(1):62-66.
3. Lopez L, Colan SD, Frommelt PC, et al. Recommendations for quantification methods during the performance of a pediatric echocardiogram: a report from the Pediatric Measurements Writing Group of the American Society of Echocardiography Pediatric and Congenital Heart Disease Council. *Journal of the American Society of Echocardiography : official publication of the American Society of Echocardiography.* 2010;23(5):465-495; quiz 576-467.
4. Eidem BW, O´Leary P. *Echocardiography in Pediatric and Adult Congential Heart Disease.* Philadelphia: Lippinkott, Wiliams & Wilkins; 2009.
5. Koestenberger M, Nagel B, Ravekes W, et al. Left ventricular long-axis function: Reference values of the mitral annular plane systolic excursion in 558 healthy children and calculation of z-score values. *Am Heart J.* 2012;164(1):125-131.
6. Nunez-Gil IJ, Rubio MD, Carton AJ, et al. Determination of normalized values of the tricuspid annular plane systolic excursion (TAPSE) in 405 Spanish children and adolescents. *Rev Esp Cardiol.*64(8):674-680.
7. Lakoumentas JA, Panou FK, Kotseroglou VK, Aggeli KI, Harbis PK. The Tei index of myocardial performance: applications in cardiology. *Hellenic journal of cardiology : HJC = Hellenike kardiologike epitheorese.* 2005;46(1):52-58.
8. Cui W, Roberson DA. Left ventricular Tei index in children: comparison of tissue Doppler imaging, pulsed wave Doppler, and M-mode echocardiography normal values. *J Am Soc Echocardiogr.* 2006;19(12):1438-1445.
9. Cattermole GN, Leung PY, Mak PS, Chan SS, Graham CA, Rainer TH. The normal ranges of cardiovascular parameters in children measured using the Ultrasonic Cardiac Output Monitor. *Critical care medicine.* 2010;38(9):1875-1881.
10. Pettersen MD, Du W, Skeens ME, Humes RA. Regression equations for calculation of z scores of cardiac structures in a large cohort of healthy infants, children, and adolescents:

© 2019 Brent B et al. *JAMA Network Open.*

an echocardiographic study. *Journal of the American Society of Echocardiography : official publication of the American Society of Echocardiography.* 2008;21(8):922-934.

11. Sadaka M, Aboelela A, Arab S, Nawar M. Electrocardiogram as prognostic and diagnostic parameter in follow up of patients with heart failure *Alexandria Journal of Medicine.* 2013;49(2):145-152.
12. Kudo Y, Yamasaki F, Kataoka H, Doi Y, Sugiura T. Effect of serum albumin on QRS wave amplitude in patients free of heart disease. *Am J Cardiol.* 2005;95(6):789-791.
13. Park MK, Guntheroth WG. *How to read pediatric ECGs.* 4th Edition ed: Mosby; 2006.
14. *Pocket book of hospital care for children: Second edition Guidelines for the management of common childhood illnesses.* Geneva: World Health Organization; 2013.
15. *Advanced Paediatric Life Support: The Practical Approach.* 3rd ed. London: BR MED J Publishing Group; 2004.
16. Goldstein B, Giroir B, Randolph A, International Consensus Conference on Pediatric S. International pediatric sepsis consensus conference: definitions for sepsis and organ dysfunction in pediatrics. *Pediatr Crit Care Med.* 2005;6(1):2-8.
17. Nir A, Lindinger A, Rauh M, et al. NT-pro-B-type natriuretic peptide in infants and children: reference values based on combined data from four studies. *Pediatr Cardiol.* 2009;30(1):3-8.
